# Temperature Abetted Synthesis of Zeolitic Imidazolate Framework-Derived 3D Zn@N–C with MXene and Gold Nanostars-Based Immunosensor for the Detection of Prostate-Specific Antigen

**DOI:** 10.1021/acsami.5c02634

**Published:** 2025-09-11

**Authors:** Rajalakshmi Sakthivel, Chia-Heng Chan, Lu-Yin Lin, Subbiramaniyan Kubendhiran, Yu-Chien Lin, Ting-Yu Liu, Ren-Jei Chung

**Affiliations:** † Department of Chemical Engineering and Biotechnology, 34877 National Taipei University of Technology (Taipei Tech), Taipei 10608, Taiwan; ‡ School of Materials Science and Engineering, 54761 Nanyang Technological University, 50 Nanyang Avenue, Singapore 639798, Singapore; § BIOBOND LTD, 1 Abacus House, Newlands Road, Corsham SN13 0BH, Wiltshire, U.K.; ∥ Department of Materials Engineering, 56082 Ming Chi University of Technology, New Taipei City 243303, Taiwan; ⊥ Department of Chemical Engineering and Materials Science, Yuan Ze University, Taoyuan City 32003, Taiwan; # High-value Biomaterials Research and Commercialization Center, National Taipei University of Technology (Taipei Tech), Taipei 10608, Taiwan

**Keywords:** prostate-specific antigen, electrochemical immunosensor, 3D Zn@N–C, MXene nanosheets, gold nanostars, differential pulse voltammetry

## Abstract

Prostate cancer (PC) is a malignant tumor that develops in the prostate cells of males and is the second most common cancer in men. Therefore, an early and accurate diagnosis of PC is crucial. Current diagnostic approaches are insufficient, highlighting an urgent need for alternative analytical platforms that target specific PC biomarkers in body fluids. With this instigation, we designed an environmentally friendly, disposable, and label-free electrochemical immunosensor based on gold nanostars decorated on ZIF-8-derived zinc with nitrogen-doped carbon at different temperatures (600–1000 °C) with a titanium carbide (MXene) nanosheet (AuNSs/Zn@N–C/MXene) composite modified with a screen-printed carbon electrode (SPCE) to detect prostate-specific antigen (PSA). Zn@N–C-800 °C with MXene possesses high conductivity and catalytic activity, a large surface area, and abundant active sites for PSA immunosensors. The AuNSs further enhanced the conductivity of Zn@N–C/MXene/SPCE. Cysteamine (Cys) and glutaraldehyde (Glut) were connected to the antibody (Ab) PSA to form an Ab/Glut/Cys/AuNSs/Zn@N–C-800 °C/MXene/SPCE immunosensor, which showed a direct relationship between the PSA concentration and the current response of the immunosensor. It has a linear range of 0.1 pg/mL to 1 μg/mL, with an *R* ^2^ = 0.993 (*n* = 5; RSD <2%), and a detection limit (LOD) of 8.48 fg/mL. The resultant immunosensor was highly selective, stable, and reproducible, making it a promising tool for the early diagnosis and treatment of PC.

## Graphical abstract

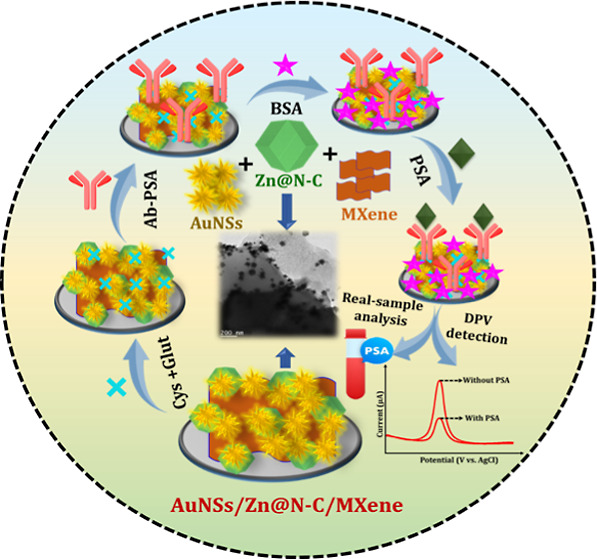

## 1 Introduction

Recent advancements in cancer biomarker analysis and proteomic research are crucial for understanding cancer biology.^1^ Prostate cancer (PC) is the most frequently diagnosed cancer in men globally and can be life-threatening.^2^ Prostate-specific antigen (PSA) is a glycoprotein with a molecular weight of 30 kDa,^3^ is secreted through the prostate gland, and is a well-known biomarker of PC when present in significant quantities in the blood. With the onset of the disease, the concentration of PSA in the blood increases from 1–4 ng mL^–1^ in normal tissue to 4–10 ng mL^–1^. A gradual upsurge in PSA concentration can signal an early cancer warning, even within the normal range.^4^ Hence, it is crucial to determine PSA levels and sensitively diagnose PC. The following technologies are commonly used to detect PSA: fluorescence assay (FIA), enzyme-linked immunosorbent assay (ELISA), radioimmunoassay (RIA), surface plasmon resonance immunoassay (SPRI), and chemiluminescence assay (CLIA).^5^ However, these methods have drawbacks, such as extended reaction time, inadequate sensitivity, and unreliable results.^6^ Electrochemical immunoassays are an emerging technology with several advantages, including high accuracy, sensitivity, affordability, ease of operation, and rapid detection.^7–10^ A key factor influencing their performance is the electrode substrate, which should provide a high surface area, excellent conductivity, and efficient antibody (Ab) immobilization while preserving the biological activity of the immobilized Abs.^11^ Nanomaterial-modified sensors are extensively utilized to enhance the electron transmission between biomolecules and electrode surfaces in biosensing applications.^12,13^

Metal–organic frameworks (MOFs) are porous materials consisting of metals and organic components. MOFs and their derivatives are extensively used as electrocatalysts owing to their adjustable porous structures, large specific surface areas, and active metal sites.^14^ Zeolitic imidazolate frameworks (ZIF-8), a subclass of MOF, are formed from 2-methylimidazole and zinc (Zn^2+^) ions and are promising materials for designing electrochemical sensors.^15^ Despite its low conductivity, the applications of ZIF-8 in electrochemical biosensing are limited.^16^ Conversely, MOF derivatives improve the electrochemical properties, enhance the electron transfer rates, and increase the number of active sites in electrochemical biosensors.^17^ During the synthesis of carbon-supported metal single-atom catalysts, the pyrolysis temperature significantly affects the local metal coordination of the resulting carbon products. Experimental evidence suggests that MN_4_ sites in N-doped carbon are thermodynamically stable and preferred over nanoparticles at low metal loadings and pyrolysis temperatures above 800 °C. At such temperatures, most Zn is reduced to its metallic state and evaporates due to its boiling point (907 °C), resulting in N-doped porous carbon that retains the morphology of the precursor and exhibits enhanced catalytic activity.^18^ MOF-derived carbon has also been explored beyond sensing, such as its use as a grease additive to enhance the tribological properties of bentone grease.^19^ However, in the field of biosensing, it remains challenging to achieve significant improvements in performance using a single catalyst. As a result, the development of composite materials with synergistic effects has emerged as a promising strategy.^20^ Recent studies have highlighted the potential of fluorescent MOF composites for sensitive and selective bioanalysis, including the detection of biomolecules, pathogens, and intracellular targets.^21^ In parallel, advancements in nanostructured materials have significantly enhanced electrochemical performance in various applications.^22^ For example, titanium nitride (TiN) meta-biosensors have shown considerable promise for the sensitive detection of prostate cancer biomarkers.^23^

In this context, two-dimensional transition metal carbides such as titanium carbide (MXene) have sparked research interest owing to their graphene-like structure, metallic conductivity, exceptional chemical stability, and hydrophilicity compared to typical electrode materials.^24^ Despite their potential, the electrochemical use of MXene is hindered by their tendency to restack owing to interactions such as hydrogen bonding and van der Waals forces between nanosheets.^25^ Carbon materials that possess a high electrical conductivity can be used as intercalating agents to overcome this challenge and improve the electrochemical performance of MXene. This allows the creation of composite materials with enhanced electrochemical performance.^26,27^ Integrating MXene with ZIF-8-derived Zn-based materials and carbon can optimize impedance matching and facilitate nanoscale heterointerface construction. MXene enhances conductivity and charge transport, while Zn and carbon improve catalytic activity and structural stability,^28^ making them excellent electrode materials for use in electrochemical sensors.^24,29^ To further improve the electrical conductivity and antibody (Ab) immobilization efficacy, Au-based nanomaterials were used.^30,31^

Nanometer-sized gold nanoparticles (AuNPs) are highly valued in various fields, owing to their admirable properties. In biosensors, AuNPs enable signal amplification in sensors because of their biocompatibility, electrical conductivity, specificity, and ease of functionalization.^7,10,32^ Various types of AuNPs have been developed, including nanorods, nanowires, nanocages, nanoshells, and nanostars (AuNSs).^33^ AuNSs with sharp branches offer strong plasmon resonance, high conductivity, easy functionalization, and excellent biocompatibility.^34–36^ Their branched tips enhance magnetic field concentration and surface area, enabling greater antibody loading and improved immunoassay performance.^37^ Therefore, they are well-suited as connectors between sensor platforms and antibodies and have numerous practical applications in biosensing.

In this study, we synthesized a AuNSs/Zn@N–C/MXene composite with stable physicochemical properties, including excellent catalytic activity, high conductivity, and a large surface area. To enable the immobilization of the PSA antibody (Ab-PSA), self-assembled monolayers (SAMs) of cysteamine (Cys) were formed on the Zn@N–C/MXene-modified electrode surface. AuNSs functionalized with thiol groups were employed to enhance the surface functionality. The cross-linking agent glutaraldehyde (Glut) was used to covalently bind the amine (−NH_2_) groups of Cys to the primary amine groups of the Ab. The resulting composite exhibited excellent conductivity and was used to construct an immunosensor for the detection of PSA in serum samples. This strategy offers a novel, eco-friendly, cost-effective, and label-free assay with promising potential for the clinical diagnosis of PC.

## 2 Experimental Sections

The Supporting Information (Sections S1–S4) delivers full details about the chemicals and reagents, characterizations, synthesis of ZIF-8, and the chlorination process of SPCE. Section S5 provides the results of the UV-Vis spectroscopy analysis.

### 2.1 Preparation of Zinc with Nitrogen-Doped Carbon (Zn@N–C)

The ZIF-8 powder was annealed in a high-temperature tube furnace. The heating rate was set to 5 °C/min, while nitrogen gas was passed through at a rate of 5 cm^3^ per min. The furnace was heated at diverse temperatures (600, 700, 800, 900, and 1000 °C) for 2 h and then allowed to cool to atmospheric temperatures. The Zn@N–C black powder was carbonized at various temperatures to obtain the products, which were used for the electrochemical sensing processes. Figure 1a shows the synthesis of ZIF-8-derived Zn@N–C via carbonization.

**Figure 1:**
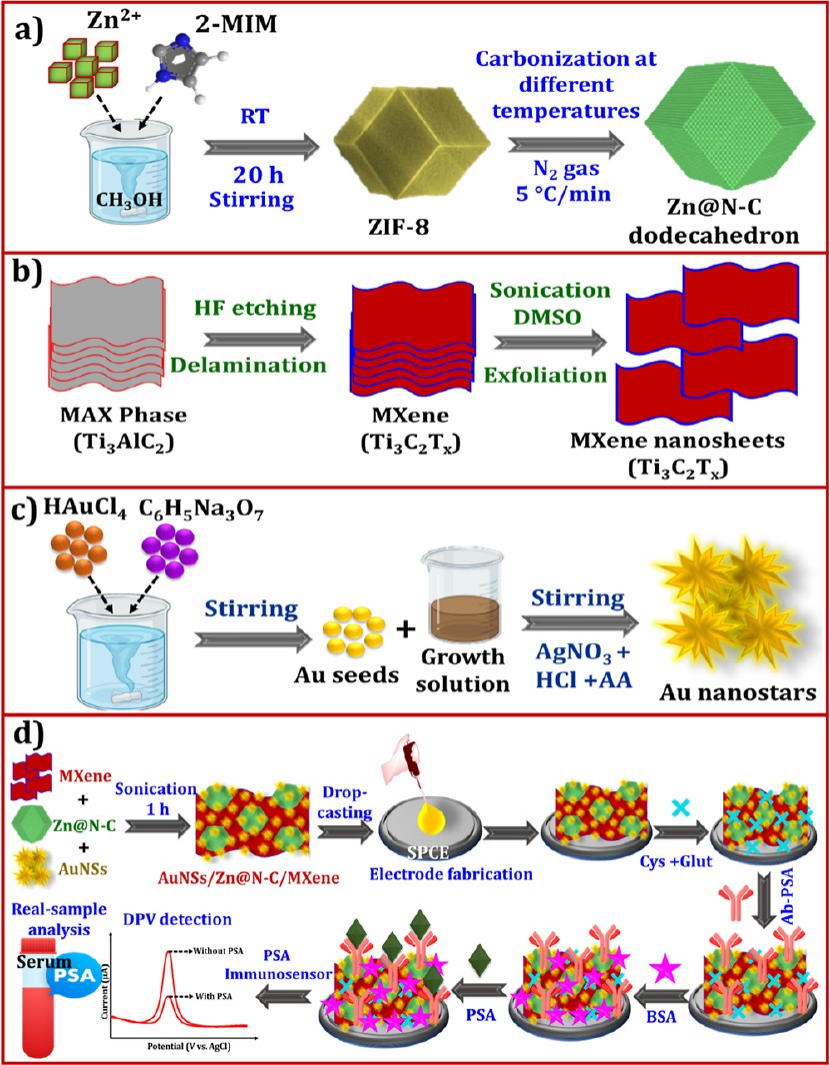
Schematic presentation of the synthesis of (a) ZIF-8-derived Zn@N–C (600–1000 °C), (b) MXene nanosheets, (c) AuNSs, and (d) fabrication of the PSA immunosensor.

### 2.2 Preparation of MXene Nanosheets

2.5 g portion of the MAX phase (Ti_3_AlC_2_) was dispersed in 50 mL of 49% HF and stirred until it was completely dispersed. The mixture was then transferred to a Teflon liner inside a hydrothermal kettle, which was then placed in an oven and fixed at 50 °C for 36 h and then allowed to cool to atmospheric temperature. After cooling, the solution and the supernatant were removed, and the residue was washed with water until the pH was above 5. The supernatant was decanted, and the solid was allowed to dry. Subsequently, 1 g of the precipitate was dissolved in 20 mL of DMSO and stirred for 18 h. Then, the solution was centrifuged at 9000 rpm for 10 min and washed once with water to collect the product. Subsequently, the MXene precipitate was dispersed in 500 mL of water and vibrated with ultrasonic waves for 6 h. It was then centrifuged at 9000 rpm for 15 min and washed twice with water, and the supernatant was removed. The resulting MXene nanosheets were then dried in a 50 °C oven for further use. The preparation of the MXene nanosheets is shown in Figure 1b.

### 2.3 Preparation of Gold Nanostars

Before the AuNSs were prepared, gold nanoseeds were prepared. Next, 2 mL of a 10 mM HAuCl_4_ solution was added to a beaker, and a 1 mM HAuCl_4_ solution was diluted to 20 mL and heated until boiling. Next, 3 mL of 1 wt % sodium citrate was added, and the solution was boiled under a stirrer for 10 min until it changed to wine-red. Next, the cooling solution was filtered to obtain the gold nanoseed solution, which was stored in a 4 °C refrigerator. A 0.25 mM HAuCl_4_ solution was prepared using a 10 mM HAuCl_4_ solution by dilution. AuNSs were prepared by the following process: 200 μL of hydrochloric acid (1 M), 2 mL of gold nanoseed solution, 2 mL of silver nitrate (3 mM), and 1 mL of ascorbic acid (0.1 M) were sequentially added one by one to the 200 mL of HAuCl_4_ (0.25 M) solution and stirred continuously for 30 s. Finally, the resultant solution was centrifuged at 4000 rpm for 15 min and filtered, and the AuNSs solution was stored in a 4 °C refrigerator for electrochemical experiments. Figure 1c shows the synthesis of the AuNSs.

### 2.4 Fabrication and Detection of PSA

The step-by-step construction process of the label-free PSA immunosensor is shown in Figure 1d. First, the AuNSs/Zn@N–C-800 °C/MXene solution, 4 mg of MXene, and 4 mg of Zn@N–C-800 °C were dispersed in 1 mL of the AuNSs aqueous solution and placed in an ultrasonic vibration tank for 10 min. DI water was used as the solvent to prepare 20 mM Cys and 0.05% Glut solutions. PBS was used as the solvent to prepare 1% BSA, 12.5 μg/mL Ab, and different concentrations of PSA antigens (0.1 pg/mL to 1 μg/mL). Six microliters of AuNSs/Zn@N–C-800 °C/MXene were dropped on the GCE and dried at 37 °C for 60 min. Six microliters of Cys and 6 μL of Glut were dropped sequentially onto the working electrode and placed at 37 °C for 40 and 50 min, respectively. Next, 6 μL of Ab-PSA was drop-cast on Glu/Cys/AuNSs/Zn@N–C-800 °C/MXene/SPCE and kept at ambient temperature for 70 min. Subsequently, 6 μL of blocking agent BSA was immobilized on the fabricated electrode and placed at room temperature for 80 min. Finally, 6 μL of PSA antigens with diverse concentrations (0.0001, 0.001, 0.01, 0.1, 1, 10, 100, 1000 ng/mL) were dropped on the BSA/Ab-PSA/Glut/Cys/AuNSs/Zn@N–C-800 °C/MXene/SPCE kept at atmospheric temperature for 120 min. CV, EIS, and DPV were used for subsequent detection. Following the complete formation of an antigen–antibody immunocomplex, the electrode was checked using DPV in 10 mL of PBS (pH 7.4; 0.1 M) containing 5 mM [Fe­(CN)_6_]^3–/4–^. The experimental parameters are as follows: potential window = −0.2 to 0.6 V, increase *E*(*V*) = 0.004, amplitude (*V*) = 0.05, pulse width = 0.05, sampling width = 0.0167, pulse period (s) = 0.5, quiet time (s) = 2, and sensitivity (*A*/*V*) = 1 × 10^–5^.

### 2.5 Actual Sample Processing

The specificity of the PSA immunosensor was evaluated by using human serum samples. Blood samples were collected from healthy men and subjected to pretreatment. The collected serum samples were diluted with PBS and divided into five portions, each containing different PSA concentrations. The samples were then individually analyzed using an electrochemical immunosensor, and the recovery was calculated using the following equation

1 $$recovery=\frac{found}{added}\times 100$$

## 3 Results and Discussion

### 3.1 Characterizations of ZIF-8-Derived Zn@N–C

FESEM is used to examine the surface characteristics of ZIF-8 at different magnifications and Zn@N–C at increasing temperatures (600–1000 °C), with diverse magnification results, and the corresponding EDX results are displayed in Figure 2. The FESEM image of ZIF-8 exhibits a dodecahedron structure, and its particle size of approximately 2 μm can be observed (Figure 2a,b).

**Figure 2:**
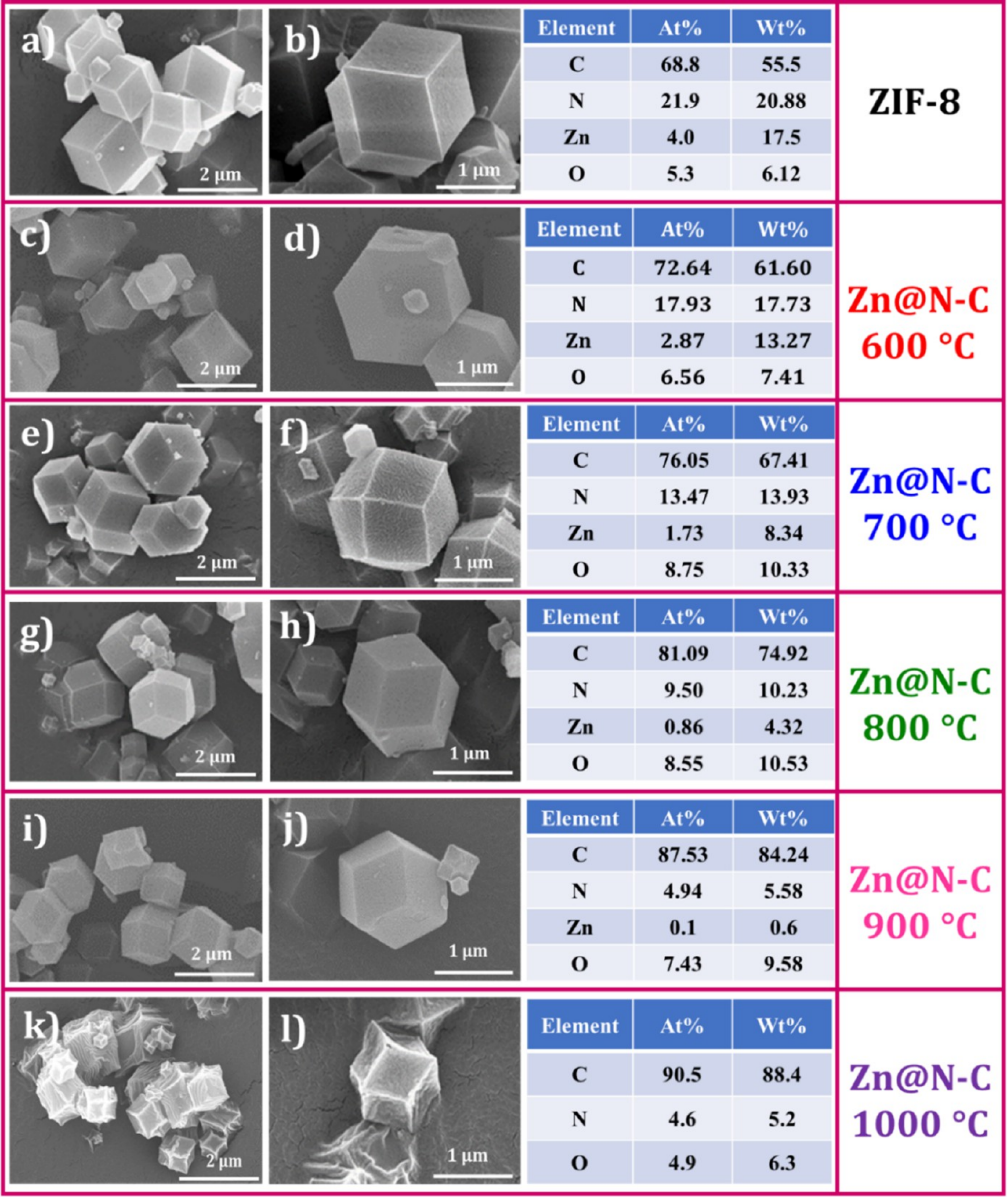
FESEM images of (a,b) ZIF-8 at different magnifications with the corresponding EDX results and (c–l) FESEM images of Zn@N–C at different magnifications with increasing temperatures (600–1000 °C) and the corresponding EDX results.

After calcination, ZIF-8 was converted to the dodecahedral structure of Zn@N–C (600–1000 °C), and the variations in the particle size became smaller as the temperature increased (600–1000 °C). However, at 1000 °C, the crystal collapsed because of the boiling point of Zn. Above 800 °C, Zn nearly completely volatilized, leading to structural collapse. Hence, 800 °C is optimum, and Zn@N–C possesses numerous pores, which can enhance the contact area for redox reactions. In addition, the EDX spectrum of ZIF-8 to Zn@N–C-(600–1000 °C) was displayed in Figure 2. The EDX analysis indicated that carbon accounted for the largest proportion of the entire material. As the calcination temperature increased, the carbon content continued to increase, whereas the nitrogen and Zn contents decreased. Even the existence of Zn was nearly undetectable in Zn@N–C-(600 to1000 °C), which supports the theory that the collapse of the structure was related to the complete volatilization of Zn. The decrease in the nitrogen content does not correlate with the oxygen content. At elevated temperatures, nitrogen-containing groups decompose, resulting in a reduction of the number of CN active sites. In contrast, the increase in oxygen detected by EDX is attributed to the incorporation of atmospheric oxygen into the material. In addition, Figure S1a–x shows the EDX elemental mappings of ZIF-8 to Zn@N–C (600–1000 °C); this result confirms the existence of carbon, nitrogen, and zinc elements in ZIF-8 and Zn@N–C at diverse temperatures (600–1000 °C).

XRD results are used to confirm the crystallinity and phase purity of the ZIF-8 and Zn@N–C-(600–1000 °C) materials, as shown in Figure 3a. As can be seen in Figure 3a inset, the characteristic peaks at 2θ values of 7.5°, 10.5°, 12.8°, 14.9°, 16.6°, 18.2°, 22.2°, 24.6°, 26.9°, and 29.8° are assigned to (011), (002), (112), (022), (013), (222), (114), (233), (134), and (044) crystal planes, consistent with the simulated pattern of ZIF-8 (JCPDS No: 00-062-1030);^38^ this finding suggests the ZIF-8 formation. Figure 3a demonstrates the XRD patterns of Zn@N–C at various calcination temperatures (600, 700, 800, 900, and 1000 °C). The original peaks of ZIF-8 vanished completely, while two broad peaks emerged at 29° and 43°, agreeing with the (002) and (101) planes of amorphous carbon,^18,39^ indicating successful carbonization of ZIF-8 into Zn@N–C (600, 700, 800, 900, and 1000 °C). At lower pyrolysis temperatures, the two main peaks were broad and not distinct, indicating a highly disordered carbon structure. With increasing pyrolysis temperature, the peaks became narrower and stronger, suggesting a more ordered carbon structure, approaching that of graphite. Although no Zn-relevant peaks were detected in the XRD patterns, the existing Zn either evaporated to a very few levels or was transformed into an amorphous state. The metallic Zn boiling point is 908 °C, suggesting that metallic Zn can be quickly reduced from Zn ions during the carbonization process. The calcination temperature significantly influenced the presence of Zn.^40^ The presence of Zn in the materials carbonized at different temperatures was confirmed by EDX, mapping, and XPS.

**Figure 3:**
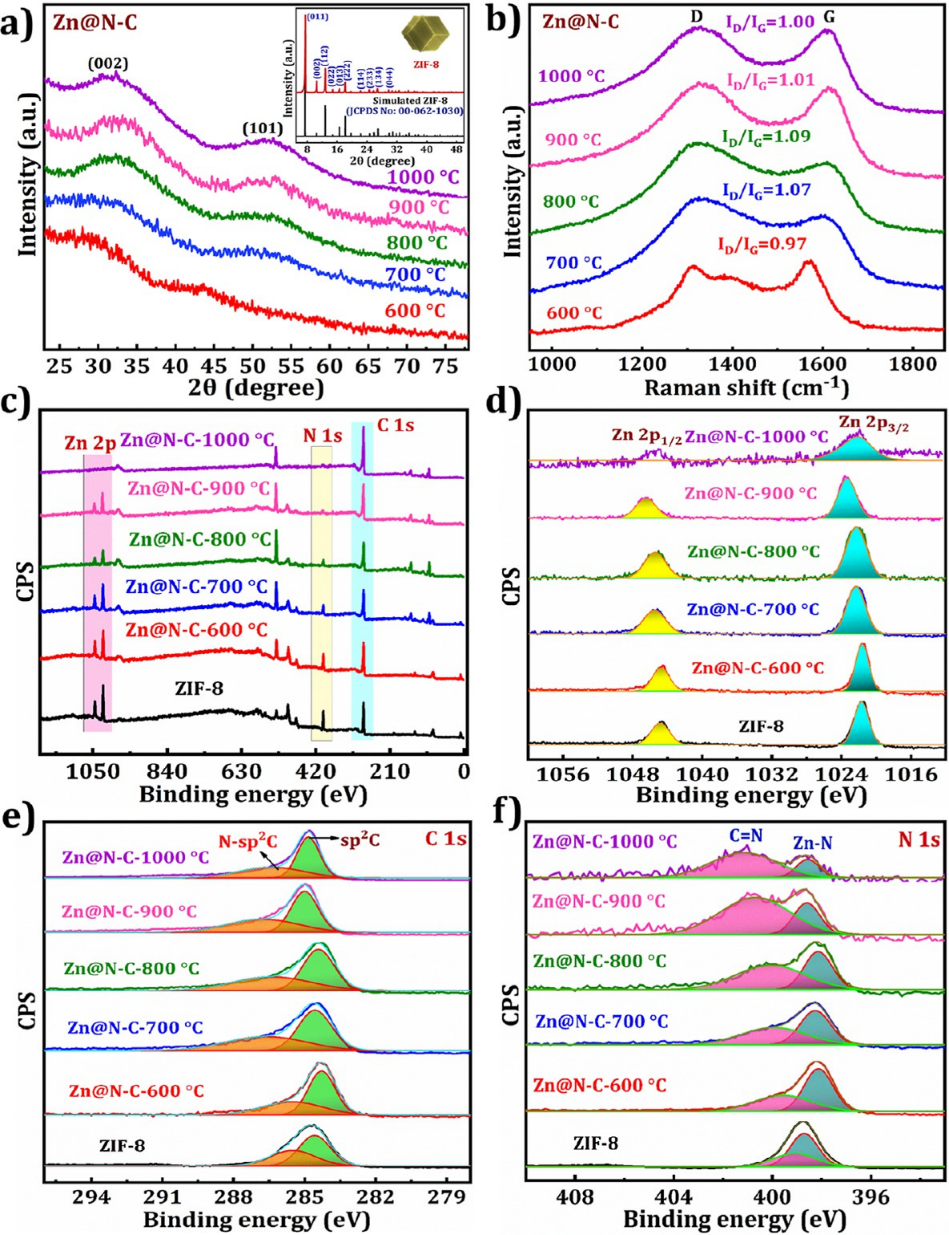
(a) XRD patterns of Zn@N–C-(600–1000 °C) and inset: XRD pattern of ZIF-8 with simulated ZIF-8. (b) Raman and (c) XPS survey spectra of Zn@N–C-(600–1000 °C) and XPS deconvoluted spectra of (d) Zn 2p, (e) C 1s, and (f) N 1s in Zn@N–C-(600–1000 °C).

The Raman spectra of Zn@N–C materials carbonized at different temperatures (600–1000 °C) were analyzed and are presented in Figure 3b. The spectra showed distinctive D and G bands at 1330 and 1580 cm^–1^, indicating the successful carbonization of ZIF-8 into Zn@N–C. From Zn@N–C-600 to Zn@N–C-1000 °C, the D band and G band intensity ratios (*I*_D_/*I*_G_) are 0.97, 1.07, 1.09, 1.01, and 1, respectively. The *I*_D_/*I*_G_ ratio increased as the calcination temperature rose from 600 to 800 °C, suggesting the presence of highly amorphous carbon without a graphite structure. However, at 900 °C, the *I*_D_/*I*_G_ ratio decreases as the benzene rings start to form a graphite structure. Above 900 °C, the *I*_D_/*I*_G_ ratio continued to decline, indicating an increase in graphitization and a decrease in structural defects.^41^ Therefore, it can be inferred that higher temperatures led to a higher graphitization of Zn@N–C.

XPS was used to qualitatively analyze the surface of the material to confirm its elemental composition and valence states. In Figure 3c, the survey spectra of ZIF-8 and Zn@N–C (600–1000 °C) are shown. Significantly, ZIF-8 and Zn@N–C (600 to 900 °C) contain Zn, O, C, and N elements. However, Zn@N–C-1000 °C does not show the characteristic peak of Zn, but a lower intensity peak is observed in the high-resolution spectrum in Figure 3d. As shown in Figure 3d, the deconvoluted peaks of 2p_3/2_ (1021.5 eV) and 2p_1/2_ (1044.6 eV) in the Zn 2p spectrum of ZIF-8 confirm the Zn^2+^ state of Zn.^16^ Compared to ZIF-8, the Zn 2p_3/2_ and Zn 2p_1/2_ peaks of Zn@N–C (600–900 °C) shift to higher binding energy, suggesting a high electron-rich state of Zn 2p in this material compared to the other.^42^ However, Zn@N–C-1000 °C shows a shift toward the lower binding energy with low-intensity peaks; this result confirms the lower content of Zn in the Zn@N–C-1000 °C material, which may be due to several factors. Some Zn species might remain trapped in the carbon matrix, preventing complete evaporation. Additionally, Zn–N or Zn–C bonds can be stable at high temperatures, leaving trace amounts in the material.

Figure 3e shows the deconvoluted spectrum of C 1s, whereas the peaks that emerged at 284.6 and 285.6 eV correspond to sp^2^C and N-sp^2^C^43^ in ZIF-8. On increasing the temperature from 600 to 1000 °C for the Zn@N–C samples, the intensity of the sp^2^C peak increased, while the intensity of the N-sp^2^C peak decreased. This change was attributed to the increase in carbon content at higher temperatures. In Figure 3f, the existence of the N 1s characteristic peaks located at 398.7 and 399.2 eV indicates the existence of the Zn–N and C=N bonds, respectively.^44,45^ In Zn@N–C-(600–1000 °C), the intensity of the Zn–N peak gradually declined and the peaks shifted toward the lower binding energy. The results aligned closely with the findings from the EDX and XRD analyses.

### 3.2 Characterizations of Zn@N–C-800 °C, MXene, AuNSs, and AuNSs/Zn@N–C-800 °C/MXene

The structural characteristics of Zn@N–C-800 °C, MXene, AuNSs, and the composite AuNSs/Zn@N–C-800 °C/MXene were investigated by using FESEM, TEM, XRD, Raman, UV–vis, and XPS analyses. The electrochemical characteristics of these materials were studied by using CV, EIS, and DPV techniques.

The FESEM and TEM images of Zn@N–C-800 °C, MXene, and AuNSs can be seen in Figure 4. After careful optimization, Zn@N–C-800 °C was chosen for the composite preparation and electrochemical immunosensor. Figure 4a shows that Zn@N–C-800 °C possesses a dodecahedral structure. To analyze the particle size of Zn@N–C-800 °C, a histogram was generated from FESEM images by using ImageJ software. Figure S2a,b presents a large-scale FESEM image along with the particle size distribution, indicating an average size of approximately 1.25 ± 0.2 μm. The surface characteristics of MXene are shown in Figures 4b, S3a, and S4a, revealing an accordion-like structure at diverse magnifications. These findings align with the theory that the Ti–Al metal bonds are weaker than Ti–C bonds. The HF selectively eliminated the Al layer to create an accordion structure. Figure S3a,b presents a large-scale FESEM image of MXene, revealing an average particle size of approximately 8.5 ± 0.5 μm. Besides, the elemental mapping images of MXene are shown in Figure S4b–f, indicating the formation of MXenes. Figure 4c and g show the FESEM and TEM images of the AuNSs, displaying star-like structures with a size of approximately 50–100 nm.

**Figure 4:**
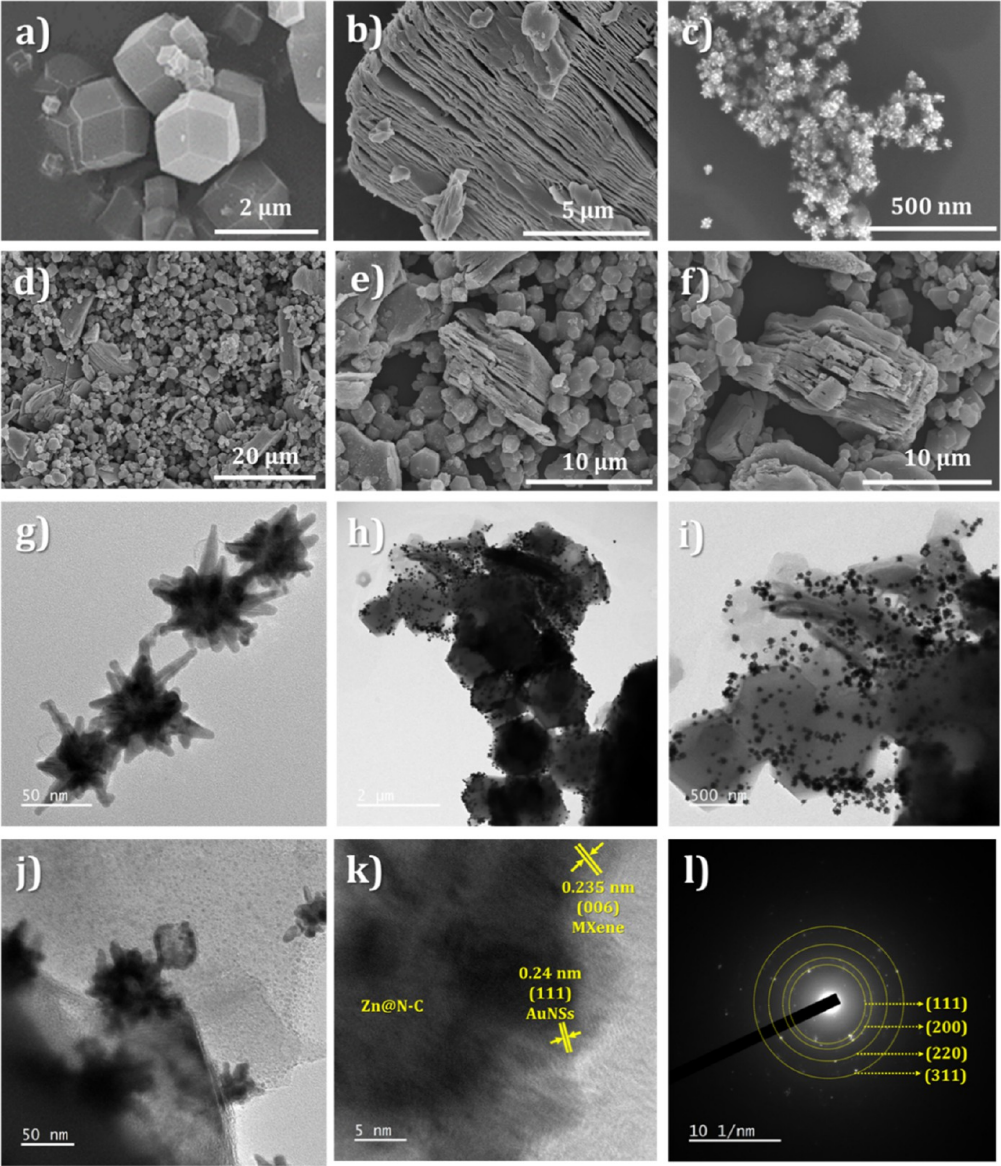
FESEM images of (a) Zn@N–C-800 °C, (b) MXene, (c) AuNSs, and (d–f) AuNSs/Zn@N–C-800 °C/MXene with diverse magnifications. TEM images of (g) AuNSs and (h–j) AuNSs/Zn@N–C-800 °C/MXene with diverse magnifications. (k) HRTEM and (l) SAED pattern of AuNSs/Zn@N–C-800 °C/MXene.

Figure 4d–f and h–j show the FESEM and TEM images of AuNSs/Zn@N–C-800 °C/MXene with diverse magnifications, revealing the even decoration of AuNSs on the dodecahedron structure of Zn@N–C-800 °C with the layered structure of MXene. The HRTEM image (Figure 4k) shows lattice fringes with *d*-spacing values of 0.235 and 0.24 nm, which are ascribed to the (006) and (111) planes designating the MXene and AuNSs. Furthermore, the HRTEM and SAED (Figure 4l) results are relevant to the XRD results, confirming the successful formation of the AuNSs/Zn@N–C-800 °C/MXene composite. The UV–vis spectrum (Figure S5) further validates the accomplished formation of AuNSs, with additional details outlined in Section S5.

The elemental composition distribution of AuNSs/Zn@N–C-800 °C/MXene was identified from elemental mappings and EDX, as presented in Figure 5a–g. As can be seen in Figure 5, the (a) AuNSs/Zn@N–C-800 °C/MXene composite (Mix) contains elements of (b) Zn, (c) C, (d) N, (e) Ti, and (f) Au. In addition, Figure 5g shows the EDX spectrum of AuNSs/Zn@N–C-800 °C/MXene, and it has the signals with weight percentages of Zn, C, N, Ti, and Au of 6.62, 35.90, 8.23, 16.10, and 33.10%, demonstrating that the AuNSs/Zn@N–C-800 °C/MXene composite was formed successfully. In addition, the chemical bonds and functional groups of MXene were identified using Raman spectroscopy, as depicted in Figure S4g. The Raman spectrum of MXene reveals that peaks at 142, 385, 502, and 623 cm^–1^ are the vibration modes of anatase, while both peaks at 1371 and 1575 cm^–1^ are the typical D and G bands of graphitic carbon,^46^ further proving the generation of MXene.

**Figure 5:**
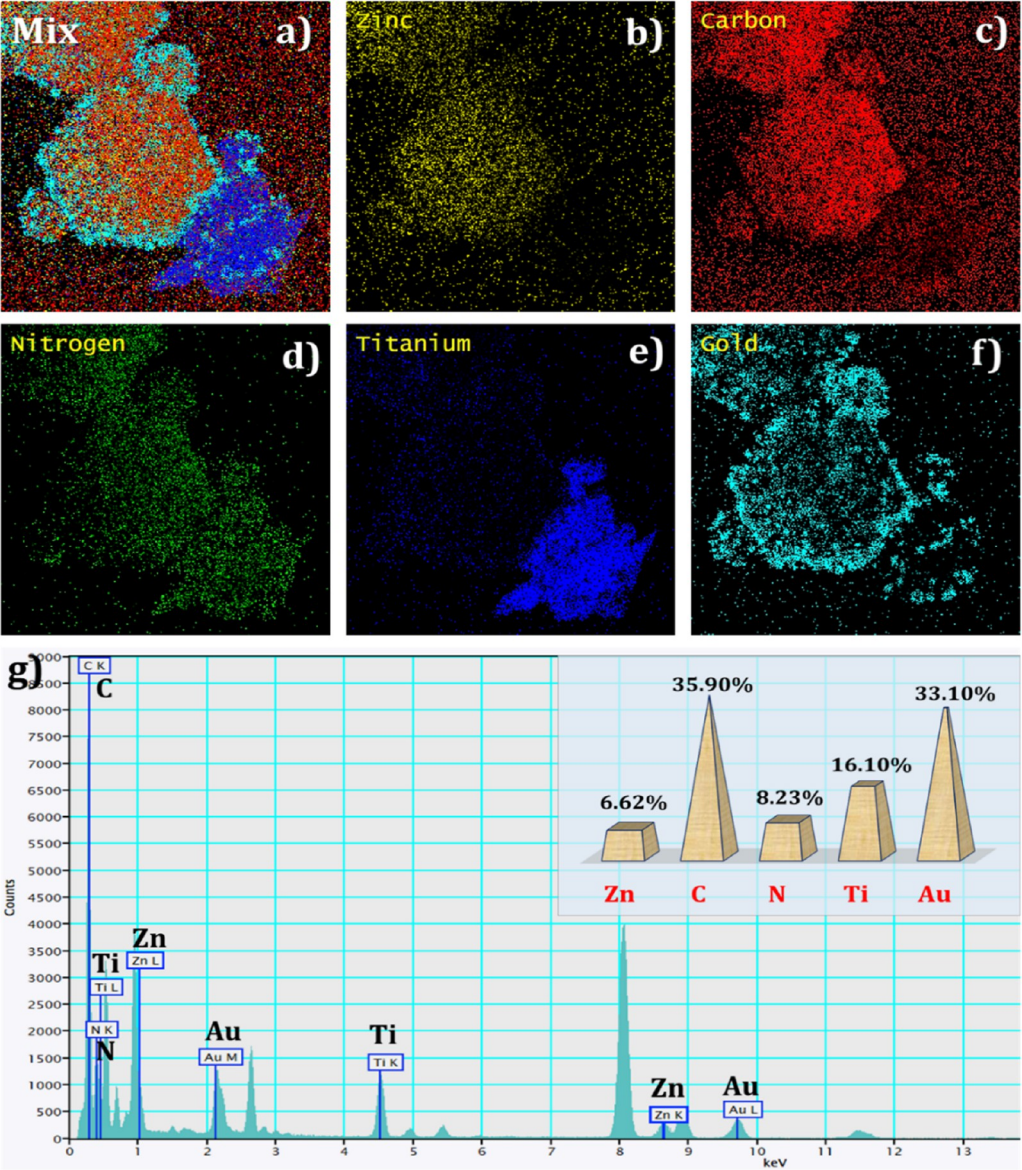
Elemental mapping of (a) AuNSs/Zn@N–C-800 °C/MXene-Mix and existing elements of (b) Zn, (c) C, (d) N, (e) Ti, and (f) Au. (g) EDX spectrum of AuNSs/Zn@N–C-800 °C/MXene.

The crystallinities and lattice structures of MXene, Zn@N–C-800 °C, and AuNSs/Zn@N–C-800 °C/MXene were investigated by using XRD, as demonstrated in Figure 6a. It can be seen that the diffractions at 8.9°, 18.3°, 27.8°, and 60.9° are attributed to (002), (006), (008), and (110) crystal planes, respectively, confirming MXene sheets formation.^47,48^ The diffraction of Zn@N–C-800 °C exhibits two broad peaks that emerged at 29° and 43°, consistent with the (002) and (101) planes of amorphous carbon.^18,39^ Additionally, the peaks at 25.1°, 38.3°, 44.4°, 64.7°, and 77.9° correspond to (202), (111), (200), (220), and (311), respectively, suggesting the existence of AuNSs with cubic symmetry consistent with previous report;^49^ besides, the diffraction peaks of MXene and Zn@N–C-800 °C reappeared in the AuNSs/Zn@N–C-800 °C/MXene, indicating successful composite formation.

**Figure 6:**
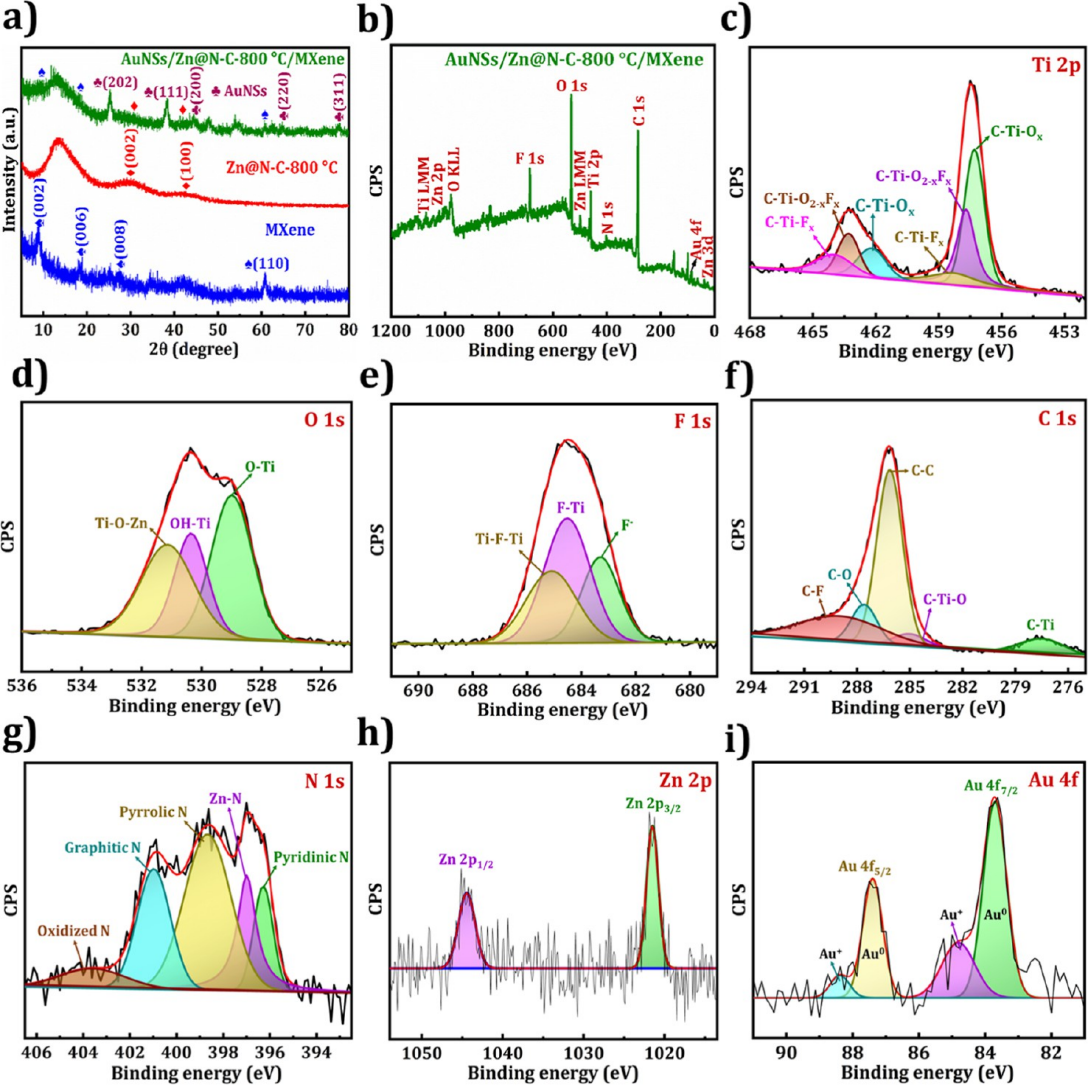
(a) XRD patterns of MXene, Zn@N–C-800 °C, and AuNSs/Zn@N–C-800 °C/MXene. XPS spectra of (b) AuNSs/Zn@N–C-800 °C/MXene (survey), (c) Ti 2p, (d) O 1s, (e) F 1s, (f) C 1s, (g) N 1s, (h) Zn 2p, and (i) Au 4f.

In Figure 6b, the survey spectrum of AuNSs/Zn@N–C-800 °C/MXene shows existing elements of Zn, Au, C, N, Ti, O, and F, and the MXene-survey spectrum (Figure S4h) show the elements C, Ti, O, and F. Significantly, the peak assignment of the Ti 2p region (Figure 6c) has six deconvoluted peaks located at 457.3, 457.7, 458.4, 462.2, 463.3, and 464 eV, which are attributed to C–Ti–O_*x*_, C–Ti–O_2‑x_F_*x*_, C–Ti–F_*x*_, C–Ti–O_*x*_, C–Ti–O_2–*x*_F_*x*_, and C–Ti–O_*x*_, respectively.^50^ The O 1s region (Figure 6d) of AuNSs/Zn@N–C-800 °C/MXene can be fitted into three peaks that emerged at 529 eV (Ti–O), 530.4 eV (Ti–OH), and 531.2 eV (Ti–O–Zn), representing the chemical bonds formation at the interface between Zn@N–C-800 °C and MXene. The interfacial bonding of the Ti–O–Zn formation is helpful to anchor Zn@N–C-800 °C with the MXene matrix tightly and strengthen the interaction between the two components.^42^ In Figure 6e, the F 1s region shows the three deconvoluted peaks at 683.2 eV (F^–^), 684.5 eV (F–Ti–C), and 685.1 eV (C–Ti–F–Ti–C), demonstrating fluorine is a bridging atom^51^ in the composite. The XPS spectrum of C 1s (Figure 6f) exhibits five peaks located at 277.5 eV (C–Ti), 285 eV (C–Ti–O), 286.1 eV (C–C), 287.6 eV (C–O), and 288.9 eV (C–F) in AuNSs/Zn@N–C-800 °C/MXene.^52^ The high-resolution N 1s spectrum (Figure 6g) can be divided into five peaks at 396.3 eV (pyridinic-N), 397 eV (Zn–N), 398.6 eV (pyrrolic-N), 401 eV (graphitic-N), and 403.6 eV (quaternary N^+^–O^–^).^53^ The presence of pyridinic and graphitic N has been widely acknowledged to augment the electrocatalytic activity of the electrocatalysts. The Zn–N bond was detected at 397 eV, indicating that the Zn atoms were indeed doped into the graphene structure and bonded with the doped N. The Zn 2p region (Figure 6h) shows deconvoluted peaks at 1021.5 and 1044.5 eV ascribed to Zn 2p_3/2_ and Zn 2p_1/2_, and it has a 2+ oxidation state.^48^ Figure 6i presents the Au 4f core-level spectrum, revealing four peaks due to spin–orbit coupling. The primary peaks at 83.7 and 87.4 eV correspond to metallic gold (Au^0^), while the smaller peaks at 84.8 and 88.4 eV indicate the presence of Au^+^ oxidation states.^54^ These results suggest AuNSs/Zn@N–C-800 °C/MXene composite formation.

The specific surface areas and pore structures of the synthesized materials were characterized by using nitrogen (N_2_) adsorption–desorption isotherms. As shown in Figure S6a, pristine ZIF-8 exhibited a high BET surface area of 1384.952 m^2^/g, indicating its microporous nature with a pore size of 1.857 nm. Upon thermal treatment at increasing temperatures (600–1000 °C), the BET surface areas of the ZIF-8 derived Zn@N–C materials changed significantly, with values of 510.803, 544.536, 588.085, 539.581, and 588.350 m^2^/g for Zn@N–C-600 °C, Zn@N–C-700 °C, Zn@N–C-800 °C, Zn@N–C-900 °C, and Zn@N–C-1000 °C, respectively (Figures S6b–f). The surface area increased with temperature, particularly between 700 and 800 °C, due to the progressive volatilization of metallic zinc, which promotes the formation of a more porous three-dimensional carbon network. The pore structure characteristics were further analyzed using Barrett–Joyner–Halenda (BJH) pore size distribution curves, as shown in the insets of Figure S6b–f). The pore sizes of Zn@N–C-600 °C, Zn@N–C-700 °C, Zn@N–C-800 °C, Zn@N–C-900 °C, and Zn@N–C-1000 °C are 1.787, 1.869, 2.490, 2.102, and 2.128 nm, respectively, indicating a shift toward mesoporous structures. Additionally, MXene and the Zn@N–C-800 °C/MXene composite exhibited BET surface areas of 8.358 m^2^/g and 556.283 m^2^/g, with pore sizes of 27.159 and 2.018 nm, respectively (Figure S6g–h), confirming their mesoporous characteristics. These results highlight the significant influence of the calcination temperature and material composition on surface area and porosity.

### 3.3 Electrochemical Characterizations of Zn@N–C-800 °C, MXene, AuNSs, and AuNSs/Zn@N–C-800 °C/MXene

The electrochemical characteristics of Zn@N–C at diverse calcination temperatures (600–1000 °C) were analyzed using CV. Figure 7a shows that the current response of Zn@N–C-modified SPCE exhibits a significant increase in redox peak current from 600 to 800 °C, followed by a decrease at 900 and 1000 °C. This is related to the degree of graphitization and the number of CN active sites. Thermal treatment below 600 °C was not considered as it may lead to incomplete decomposition of ZIF-8, insufficient carbonization, and the presence of organic residues. Additionally, the electrochemical performance decreased at temperatures below 800 °C, with the material synthesized at 600 °C exhibiting the poorest performance. Based on the analysis, Zn@N–C-800 °C exhibited the highest current response and lowest peak-to-peak separation and was used as the modified electrode material for the experiment. In addition, Figure 7b shows the CV responses of SPCE and was modified by diverse materials, including MXene, Zn@N–C-800 °C, Zn@N–C-800 °C/MXene, and AuNSs/Zn@N–C-800 °C/MXene composite material. As can be seen in Figure 7b, the redox peak current responses gradually increased after modifying with MXene, Zn@N–C-800 °C, Zn@N–C-800 °C/MXene, and AuNSs/Zn@N–C-800 °C/MXene compared to bare SPCE. Due to the significant synergistic effect (larger surface area, higher catalytic activity, greater active sites, and superior conductivity) between MXene, Zn@N–C-800 °C, and AuNSs, it inclined the electron transfer rate in the electrochemical reaction.

**Figure 7:**
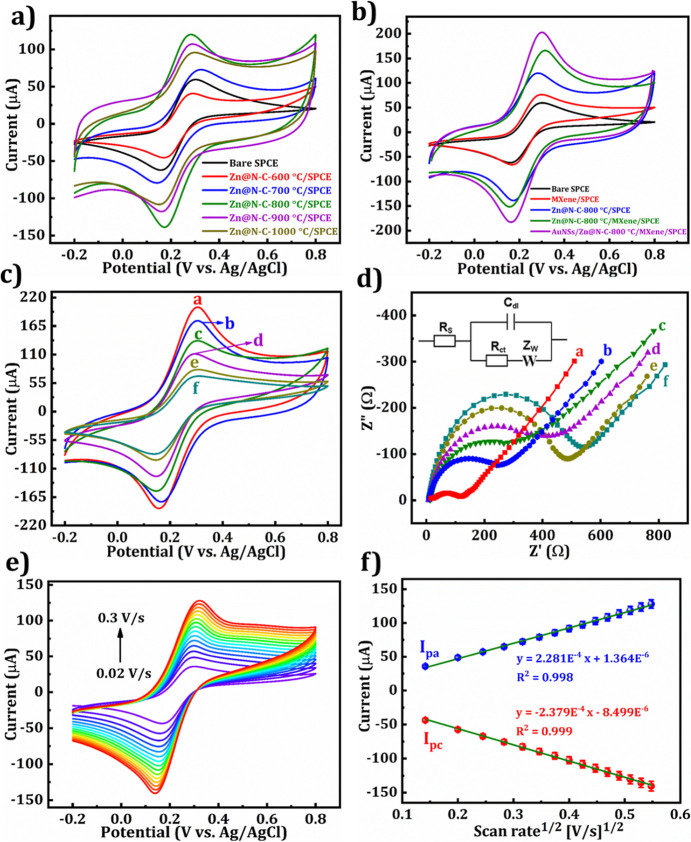
(a) CV responses of Zn@N–C at different calcination temperatures (600–1000 °C) material on SPCE and bare SPCE, and (b) CV curves of bare SPCE, MXene/SPCE, Zn@N–C-800 °C/SPCE, Zn@N–C-800 °C/MXene/SPCE, and AuNSs/Zn@N–C-800 °C/MXene/SPCE. (c) CV and (d) EIS results of a-AuNSs/Zn@N–C-800 °C/MXene/SPCE, b-Cys/AuNSs/Zn@N–C-800 °C/MXene/SPCE, c-Glut/Cys/AuNSs/Zn@N–C-800 °C/MXene/SPCE, d-Ab-PSA/Glut/Cys/AuNSs/Zn@N–C-800 °C/MXene/SPCE, e-BSA/Ab-PSA/Glut/Cys/AuNSs/Zn@N–C-800 °C/MXene/SPCE, and f-PSA/BSA/Ab-PSA/Glut/Cys/AuNSs/Zn@N–C-800 °C/MXene/SPCE. (e) Scan rate (0.02 to 0.3 V/s) analysis of PSA/BSA/Ab-PSA/Glut/Cys/AuNSs/Zn@N–C-800 °C/MXene/SPCE with the (f) linear plot [Electrolyte: 5 mM [Fe­(CN)_6_]^3–/4–^ with 0.1 M KCl (pH 7.4, 0.1 M PBS) and scan rate: 0.05 V/s].

### 3.4 Optimization of the Experimental Parameters

To improve the sensitivity of the AuNSs/Zn@N–C-800 °C/MXene-based PSA immunosensor, we needed to optimize certain operating parameters. This included optimizing the concentrations of Cys and Ab-PSA, as well as the modification time for Cys, Glut, Ab-PSA, BSA, and PSA. The temperature and pH of the electrolyte should also be considered. The results of these optimizations are presented in Figure S7.

Cys and Glut are commonly used to link Au and Ab in biosensors. Cys possesses two functional groups: thiol (−SH) and primary amine (−NH_2_). The −SH group forms stable covalent bonds with gold (Au–SH), whereas Glut acts as a cross-linking agent, forming amide bonds with Cys and antibodies. The graph (Figure S7a) shows current changes at Cys concentrations (5–40 mM). As the concentration increased from 5 to 20 mM, the highest peak current was observed at 20 mM. However, when the concentration exceeded 20 mM, the electrode surface was excessively covered with Cys, hindering electron transfer and reducing the current signal. In addition, Figure S7b shows the current changes during Cys modification over time from 20 to 60 min. The highest current response is observed after 40 min. If the modification time is too short, Cys does not fully combine with the electrode surface. However, if the modification time is too long, Cys may aggregate and hinder electron transfer. Therefore, the optimal Cys concentration and modification time were 20 mM and 40 min, respectively. Figure S7c shows the changes in the current at different times during the modification of Glut from 20 to 70 min. The graph indicates that the highest current was observed after 50 min. If the modification time is too long, Glut may continue to cross-link, hindering electron transfer and reducing current. Therefore, 50 min was selected as the optimal Glut modification time.

To investigate the impact of Ab concentration and modification time on the electrode surface, we analyzed the optimal modification conditions for Ab. In Figure S7d, the current changes at Ab modification concentrations (2.5 to 15 μg/mL) are shown. As the concentration increases, the current value decreases until it stabilizes at 12.5 μg/mL, indicating that Ab grafting has reached saturation. Additionally, Figure S7e shows the changes in the current at different Ab modification times (30–90 min). The current value increased with modification time until it stabilized after 70 min, suggesting that Ab was cross-linked with Glut. Thus, the optimal Ab concentration is 12.5 μg/mL, and the time is 70 min.

In the biosensor experiment, the antibody is specifically bound to the antigen (Ag). However, Ag also binds nonspecifically to the electrode surface, causing detection errors. To address this issue, BSA was used to block nonspecific sites. The effectiveness of BSA was confirmed by treating the electrode surface with 1% BSA for different durations and observing the resulting changes in current, as shown in Figure S7f. The current changes during BSA modification occurred between 40 and 100 min. The longer the modification time, the lower the current until it stabilized at 80 min, indicating the saturation of nonspecific sites. Therefore, the optimal BSA modification time was 80 min.

The Ag modification time required for the immune reaction between Ag and Abs was determined. Figure S7g illustrates the changes in current at different PSA modification times (80–140 min). The current increased over time until the reaction time reached 120 min, at which point the current stabilized. This indicated that PSA was bound to Ab–PSA, making 120 min the optimal time.

The stability of the constructed PSA immunosensor was confirmed by evaluating its performance at various temperatures and pH levels. Figure S7h shows the current changes of the sensor at temperatures ranging from 4 to 40 °C, revealing that the best current response occurs at 25 °C. Extreme temperatures can cause the Ab and Ag activity to deteriorate; therefore, it is best to measure the PSA sensor at 25 °C. To assess the effects of the PSA sensor at different pH values, the electrolyte was adjusted to different pH values to test the immunosensor. Figure S7i shows the current changes in the PSA sensor at different pH values (ranging from 6.0 to 8.5), revealing that the highest current response occurred at pH 7.4, which was the selected electrolyte.

### 3.5 Layer-by-Layer Construction of the PSA Immunosensor

The immunosensor construction process and layer modification were confirmed by using CV and EIS analyses. The results of the diverse fabricated electrodes, namely, a-AuNSs/Zn@N–C-800 °C/MXene/SPCE, b-Cys/AuNSs/Zn@N–C-800 °C/MXene/SPCE, c-Glut/Cys/AuNSs/Zn@N–C-800 °C/MXene/SPCE, d-Ab-PSA/Glut/Cys/AuNSs/Zn@N–C-800 °C/MXene/SPCE, e-BSA/Ab-PSA/Glut/Cys/AuNSs/Zn@N–C-800 °C/MXene/SPCE, and f-PSA/BSA/Ab-PSA/Glut/Cys/AuNSs/Zn@N–C-800 °C/MXene/SPCE were examined by CV and EIS, as shown in Figure 7c and d. As observed from Figure 7c, the CV curve of AuNSs/Zn@N–C-800 °C/MXene/SPCE (a, red curve) has the highest redox peak current owing to its excellent conductivity, large surface area, more active sites, and superior electrocatalytic ability. Subsequent modifications of 20 mM of Cys (b, blue curve), 0.05% of Glut (c, green curve), 12.5 μg/mL of Ab-PSA (d-violet curve), 1% BSA (e, dark yellow curve), and 10 ng/mL of PSA (f, dark cyan curve) caused a gradual decline in the current response owing to the improved resistance to electron movement. Additionally, the protein molecules made an insulating layer when electron conductance was obstructed.^30^

EIS is a powerful technique for analyzing electron transport properties. Figure S8 presents the EIS results for different electrodes: bare SPCE, MXene/SPCE, Zn@N–C-800 °C/SPCE, Zn@N–C-800 °C/MXene/SPCE, and AuNSs/Zn@N–C-800 °C/MXene/SPCE. The bare SPCE shows the largest semicircle, indicating high resistance and poor conductivity. Modification with MXene and Zn@N–C-800 °C gradually reduced the semicircle, demonstrating improved electron transfer. The combination of MXene and Zn@N–C-800 °C further lowers the resistance due to their high surface area and catalytic activity. Notably, the AuNSs/Zn@N–C-800 °C/MXene/SPCE electrode exhibits the smallest semicircle, confirming the lowest resistance and fastest electron transfer, attributed to the excellent conductivity of the AuNSs. In addition, Figure 7d shows the EIS results for the layer-by-layer construction of the PSA immunosensor. The AuNSs/Zn@N–C-800 °C/MXene/SPCE (red curve) has the smallest semicircle with charge transfer resistance, suggesting good conductivity and noteworthy electron transfer kinetics between redox pairs. After modification with 20 mM Cys (b, blue curve), 0.05% Glut (c, green curve), 12.5 μg/mL Ab-PSA (d, violet curve), 1% BSA (e, dark yellow curve), and 10 ng/mL PSA (f, dark cyan), the charge resistance increased gradually. Significantly, the interactions between Ab–PSA and PSA immunocomplexes exhibited a high charge transfer resistance. This is because the production of immune complexes can significantly impede electron transmission to the electrode during PSA detection. The CV and EIS results indicated successful modification at each step.

### 3.6 Mechanisms of the PSA Immunosensor

Zn@N–C-800 °C demonstrates efficient electrocatalytic activity, likely due to the remarkable intrinsic properties of atomically dispersed Zn/N active sites and the enhanced diffusion provided by its porous carbon structure. This configuration not only improves the loading capacity of the sensing interface but also facilitates effective electron transfer, thereby enhancing the sensitivity and stability of the biosensor. MXene exhibits excellent hydrophilicity, electrical conductivity, and biocompatibility, making it an ideal platform for immobilizing biomolecules and promoting direct electron transfer in electrochemical sensors. The combination of Zn@N–C-800 °C and MXene in our immunosensing system introduces a synergistic effect that enhances electrochemical signal transduction. However, the Zn@N–C-800 °C/MXene material surface does not have an Ab binding site; the immobilization of Ab-PSA requires the formation of self-assembled monolayers (SAMs) of Cys on the AuNSs/Zn@N–C-800 °C/MXene/SPCE surface. In this study, Cys was used to bind AuNSs via thiol (Au-SH bond) functionalization. Glut is a cross-linking agent used to activate the amine (−NH_2_) groups present on Cys to bind with other amino ends on the Ab-PSA molecules^55^ through cross-linking chemistry. Subsequently, the constructed electrode was modified with BSA to block the nonspecific functionalities of the active sites. Finally, PSA specifically adsorbs Ab–PSA via noncovalent interactions. During the immobilization of PSA on BSA/Ab-PSA/Glut/Cys/AuNSs/Zn@N–C-800 °C/MXene/SPCE, an immunocomplex and developed protein layers were created, which led to the electron transfer blockage from reaching the sensor surface, resulting in a drop in the current response.

### 3.7 Analysis of Different Scan Rates

The kinetics of the constructed PSA/BSA/Ab-PSA/Glut/Cys/AuNSs/Zn@N–C-800 °C/MXene/SPCE immunoassay was investigated by CV at diverse scan rates. The CV analysis of PSA/BSA/Ab-PSA/Glut/Cys/AuNSs/Zn@N–C-800 °C/MXene/SPCE in the 5 mM [Fe­(CN)_6_]^3–/4–^ with 0.1 M KCl (pH 7.4), 0.1 M PBS with a scan rate (from 0.02 to 0.3 V/s) as shown in Figure 7e. As the scanning rate augmented, the peak current response was enhanced linearly, whereas the peak redox potential diverged slightly. Figure 7f illustrates that the square root of the scan rates is proportional to the redox peak currents, and the *R* ^2^ values of the oxidation and reduction linearities are 0.998 and 0.999, respectively. The constructed immunosensing system for PSA was diffusion-controlled. This finding suggests that the protein molecules were effectively immobilized on the resultant electrode surface.^11^

In addition, the electrochemically active surface area (A) of PSA/BSA/Ab-PSA/Glut/Cys/AuNSs/Zn@N–C-800 °C/MXene/SPCE was checked using the Randles–Sevcik equation^8^

2 $$i_{p}=2.69\times 10^{5}ACn^{3/2}{\left(Dv\right)}^{1/2}$$

where *i*_p_: peak current, *A*: electrode area (cm^2^), *C*: reactant concentration (mol/cm^3^), *n*: number of electrons transferred in the reaction, *D*: diffusion coefficient (cm^2^/s), and υ: scan rate (V/s). The A of PSA/BSA/Ab-PSA/Glut/Cys/AuNSs/Zn@N–C-800 °C/MXene/SPCE was assessed to be 0.081 cm^2^. The rate-determining step of the PSA immunosensor is linked to the diffusion process, and electrons remain stable during transfer.

### 3.8 Determination of PSA with the BSA/Ab-PSA/Glut/Cys/AuNSs/Zn@N–C-800 °C/MXene/SPCE Immunosensor

DPV is a more sensitive and rapid analytical technique than conduction or potentiometry. To detect PSA, DPV analysis was conducted under the optimized conditions. Figure 8a shows the DPV responses as the PSA concentration increased from 0.1 pg/mL to 1 μg/mL, while a decline in current was observed. The electrode surface was blocked by excess PSA antigen, which hindered electron transfer. The noncovalent interactions between Ab-PSA and PSA were strong, leading to the formation of an insulating layer on the electrode surface. This resulted in repulsive electrostatic interactions between the [Fe­(CN)_6_]^3–/4–^ solution and PSA.^8,11^ Figure 8b illustrates a linear graph between the log of PSA concentrations and the current response, which can be expressed as

3 I(μA)=−7.27×10−6LogCPSA(ngmL)+8.5×10−5⁣R2=0.993

**Figure 8:**
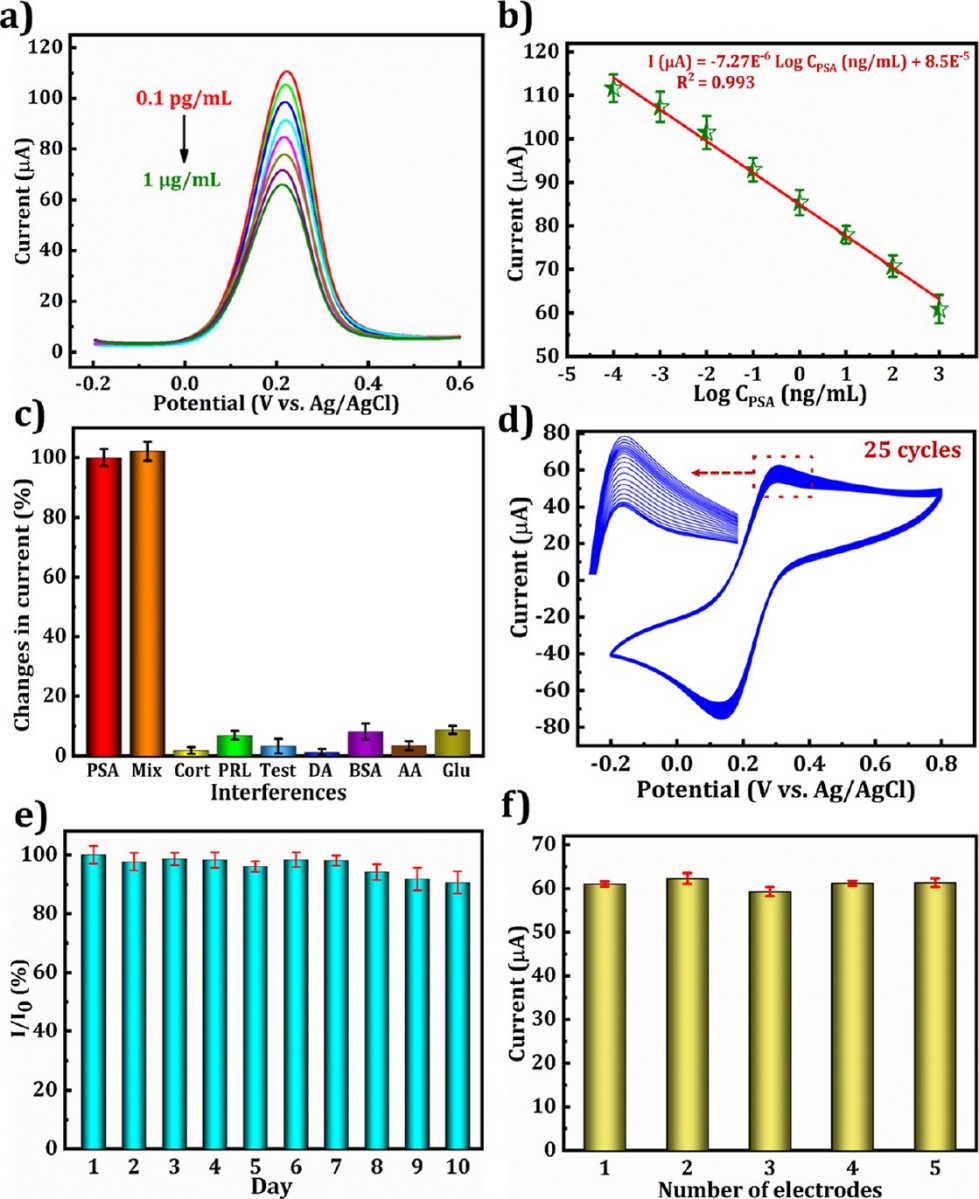
(a) PSA/BSA/Ab-PSA/Glut/Cys/AuNSs/Zn@N–C-800 °C/MXene/SPCE immunosensor with increasing concentrations of PSA (0.1 pg/mL–1 μg/mL) and the (b) corresponding linear graph. (c) Selectivity, (d) cyclic stability, (e) storage stability, and (f) reproducibility analyses results.

The calculated limit of detection (LOD) is 8.48 fg/mL based on the subsequent equation: LOD = 3.3 × σ/S, where σ and S specify the standard deviation of the current response and slope of the calibration curve.^16^ The high sensitivity of the immunosensor is due to the large surface area, low background current density, good conducting ability, biocompatibility, and catalytic activity of the AuNSs/Zn@N–C-800 °C/MXene/SPCE platform. Additionally, the strong cross-linking activity between AuNSs, Cys, Glut, and biomolecules enhanced the immobilization of Ab-PSA, leading to improved stability and amplified electrochemical signals in the constructed PSA immunosensor. Compared with a previously reported PSA immunosensor (Table S1), the proposed fabricated PSA immunosensor demonstrated significant performance, including wide linearity, the lowest LOD, and good selectivity and stability, indicating its potential for widespread application in clinical diagnosis.

### 3.9 Selectivity, Stability, and Reproducibility Analyses

To confirm the selectivity of the BSA/Ab-PSA/Glut/Cys/AuNSs/Zn@N–C-800 °C/MXene/SPCE immunosensor was employed to examine the PSA (10 ng/mL) in a solution comprising 1000 ng/mL of potential interfering substances, including cortisol (Cort), prolactin (PRL), testosterone (Testo), dopamine (DA), BSA, vitamin C (Ascorbic acid, AA), and glucose (Glu) were mixed with PSA and tested individually (Figure 8c). The current changes caused by other interferers were less than 9% when compared with PSA alone. The PSA antibody selectively reacted more with the PSA antigen than with other proteins or interferents, indicating that the PSA sensor revealed good specificity.

To ensure the stability of the PSA immunosensor, we conducted cycle and storage stability tests. After 25 cycles of CV detection, the current retention rate was 91%, as presented in Figure 8d. This indicates that the PSA immunosensor exhibits a high stability. To further confirm its stability, we stored the electrode at 4 °C for 10 days before conducting measurements. The results, presented in Figure 8e, show a 90.6% retention rate after 10 days, which is only 9.4% different from the initial measurement, demonstrating good cyclic and storage stabilities. In addition, we conducted a reproducibility test by fabricating five different PSA immunosensors using the same procedure, as shown in Figure 8f. Each sensor underwent five repeated measurements with a PSA concentration of 10 ng/mL, and the results indicated a relative standard deviation (RSD) value of 1.8%. This suggested that the constructed PSA immunosensor exhibited a high reproducibility.

### 3.10 Real Sample Analysis

The practicality of PSA was examined in human serum samples, and the tests were conducted using two diverse techniques, namely, enzyme immunoassay (ELISA) and an electrochemical immunosensor. Tables 1 and 2 present the results of the detection of PSA concentrations in real samples by using electrochemical immunosensing and ELISA, respectively. Electrochemical immunosensing demonstrated recovery rates ranging from 90.6% to 117.2% when PSA concentrations of 250–1000 pg/mL were spiked into serum samples, with an error range within 17%. In comparison, ELISA showed recovery rates between 116.4% and 129.2% for the same PSA concentration range, with an error margin of 29%. These results indicate that electrochemical immunosensors have higher accuracy and are more viable for analyzing real samples than ELISA.

**Table 1:** Detection of PSA in Human Serum with the Electrochemical Immunosensor (*n* = 5)

analyzed samples	add (pg/mL)	found (pg/mL)	recovery (%)	RSD	variance (s^2^, × 10^–11^)	*F*-test	*t*-test	d*f*
serum 1	250	282	112.8	4.87	1.88	0.81	0.44	8
serum 2	500	453	90.6	5.52	2.33	0.79	0.83	8
serum 3	1000	1172	117.2	6.42	2.94	0.64	1.28	8

**Table 2:** Detection of PSA in Human Serum with ELISA (*n* = 5)

analyzed samples	add (pg/mL)	found (pg/mL)	recovery (%)	RSD	variance (s^2^, × 10^–11^)	*F*-test	*t*-test	d*f*
serum 1	250	291	116.4	7.98	4.1	0.52	–6.13	8
serum 2	500	604	120.8	8.04	7.1	0.59	–9.64	8
serum 3	1000	1292	129.2	6.66	14.2	0.31	–15.72	8

## 4 Conclusions

This study constructed a label-free electrochemical immunosensor using the AuNSs/Zn@N–C-800 °C/MXene platform for the sensitive detection of PSA. Initially, ZIF-8-derived Zn@N–C was prepared by using various annealing temperatures ranging from 600 to 1000 °C. Zn@N–C-800 °C showed better performance than other temperatures. Following this, MXene and AuNSs were modified with Zn@N–C-800 °C. The resulting AuNSs/Zn@N–C-800 °C/MXene exhibited enhanced electrochemical characteristics, including a larger surface area, higher conductivity, biocompatibility, catalytic ability, and larger active sites. Cys was then immobilized on AuNSs/Zn@N–C-800 °C/MXene by using cross-linking chemistry with Glut. Various experimental variables, such as the concentrations of Cys and Ab-PSA and the modification times for Cys, Glut, Ab-PSA, BSA, and PSA, were optimized. The constructed PSA/BSA/Ab-PSA/Glut/Cys/AuNSs/Zn@N–C-800 °C/MXene/SPCE immunosensor has a linearity range of 0.1 pg/mL to 1 μg/mL, with a LOD of 8.48 fg/mL. Furthermore, a selectivity test confirmed that the resultant immunosensor was not affected by other biomolecules owing to its specific binding ability, and it exhibited appreciable cyclic and storage stability as well as reproducibility. The feasibility of the immunosensor was successfully demonstrated in human serum samples and compared with ELISA. In the future, this immunosensing platform will be valuable in the biomedical field for monitoring various tumor markers at the point of care and for diverse biosensing applications.

## Supplementary Material



## Notes

Chemicals and reagents; characterizations; synthesis of zeolitic imidazolate framework (ZIF-8); chlorination process of SPCE; ultraviolet–visible spectroscopy (UV–vis) analysis, elemental mapping of ZIF-8 and Zn@N–C at diverse temperatures; large-scale FESEM images with particle size distribution diagrams of Zn@N–C 800 °C and MXene; FESEM, elemental mapping, Raman, and XPS spectrum of MXene; UV–vis spectrum of AuNSs; BET analysis; optimization results of the cysteine, glutaraldehyde, antibody-PSA, bovine serum albumin, and antigen PSA, and different temperature and pH; EIS results of the unmodified and modified electrodes; and comparison of different PSA immunosensors (PDF)
