# Supplementary material for: Temperature Abetted Synthesis of Zeolitic Imidazolate Framework-Derived 3D Zn@N–C with MXene and Gold Nanostars-Based Immunosensor for the Detection of Prostate-Specific Antigen
Source: ACS Appl Mater Interfaces. 2025 Sep 11;17(38):53081–95. doi: 10.1021/acsami.5c02634 (PMC12464913; doi:10.1021/acsami.5c02634)
Supplement: Supplementary file 1 [file am5c02634_si_001.pdf]

Supporting Information

Temperature Abetted Synthesis of Zeolitic Imidazolate Framework Derived 3D Zn@N-C With MXene and Gold Nanostars-Based Immunosensor For the Detection of Prostate Specific Antigen

Rajalakshmi Sakthivel [1,*], Chia-Heng Chan [1], Lu-Yin Lin [1], Subbiramaniyan Kubendhiran [1], Yu-Chien Lin [2,3], Ting-Yu Liu [4,5,*], Ren-Jei Chung [1,6,*]

[1] Department of Chemical Engineering and Biotechnology, National Taipei University of Technology (Taipei Tech), Taipei 10608, Taiwan

[2] School of Materials Science and Engineering, Nanyang Technological University, 50 Nanyang Avenue, Singapore 639798, Singapore

[3] BIOBOND LTD, 1 Abacus House, Newlands Road, Corsham, Wiltshire, United Kingdom, SN13 0BH

[4] Department of Materials Engineering, Ming Chi University of Technology, New Taipei City 243303, Taiwan

[5] Department of Chemical Engineering and Materials Science, Yuan Ze University, Taoyuan City 32003, Taiwan

[6] High-value Biomaterials Research and Commercialization Center, National Taipei University of Technology (Taipei Tech), Taipei 10608, Taiwan

* Corresponding Author: Dr. Ren-Jei Chung

Email: rjchung@mail.ntut.edu.tw; Tel: (886-2) 2771-2171 ext 2547

Also corresponding to Prof. Rajalakshmi Sakthivel (rajalakshmi@ntut.edu.tw) and Prof. Ting-Yu Liu (tyliu0322@gmail.com)

Address: Department of Chemical Engineering and Biotechnology, National Taipei University of Technology (Taipei Tech), No. 1, Sec. 3, Zhongxiao E. Rd., Taipei 10608 Taiwan

Contents

S1. Chemicals and reagents

S2. Characterizations

S3. Synthesis of zeolitic imidazolate framework (ZIF-8)

S4. Chlorination process of SPCE

S5. Ultraviolet-visible spectroscopy (UV-Vis) analysis

Number of Figures: 08

Number of Tables: 01

Number of Pages: 16

S1. Chemicals and reagents

Zinc nitrate ($Zn(NO_3)_2{\cdot}6H_2O$), trisodium citrate ($Na_3C_6H_5O_7$), and sodium hydroxide (NaOH) were purchased from SHOWA/ Japan. Titanium aluminum carbide (Ti_3AlC_2), hydrofluoric (HF) acid, dimethyl sulfoxide (DMSO), 2-methylimidazole (2-MIM), absolute ethanol (C_2H_5OH, 99.8%), tetrachloroauric acid ($HAuCl_4$), hydrochloric acid (HCl), ascorbic acid (AA), L-cysteamine (Cy), sodium chloride (NaCl), potassium hexacyanoferrate ($K_3[Fe(CN)_6]$), potassium ferrocyanide ($K_4Fe(CN)_6$), and bovine serum albumin (BSA) were purchased from Sigma-Aldrich / USA. Methanol (CH_3OH; Fisher Chemicals /USA), silver nitrate ($AgNO_3$; Fluka Honeywell/USA), glutaraldehyde (Glut; Alfa Aesar/ USA), potassium chloride (KCl; J.T Baker/USA), sodium dihydrogen phosphate (NaH_2PO_4; J.T Baker/USA), potassium dihydrogen phosphate (KH_2PO_4; J.T Baker/USA), PSA antibody (MyBioSorse/USA), PSA antigen (Affinity biosciences/USA), and PSA ELISA Kit (Cusabio/USA) were purchased. Three-pole electrode (SPEs) were purchased from Zensor (USA) (Model TE-100).

S2. Characterizations

The morphological characteristics of the prepared samples were studied by field-emission scanning electron microscopy (FE-SEM, Regulus-8100, Hitachi, USA) and transmission electron microscopy (TEM, JEM 2100 F; JEOL Ltd., Tokyo, Japan). The elemental weight percentages of the resulting materials were determined using energy-dispersive X-ray spectroscopy (EDX). The crystalline nature and phase purity of the synthesized composite materials were analyzed using X-ray powder diffraction (XRD, X'Pert3 Powder, Malvern Panalytical, UK). Functional groups and chemical bonding of the composite material were qualitatively analyzed using Fourier-transform infrared (FTIR) spectrometry (Spotlight 200i Sp2 with AutoATR System, Perkin Elmer, USA), Raman spectroscopy (ACRON UniNanoTech Co., Ltd.), and X-ray photoelectron spectroscopy (XPS, JEOL Ltd., JPS-9030). Electrochemical experiments were performed on a CHI6114E workstation (CH Instruments, USA) equipped with a conventional three-electrode system. A screen-printed electrode (SPE, 0.07 cm^2 working area), silver/silver chloride (Ag/AgCl), and platinum filaments were used as the working, reference (auxiliary), and counter electrodes, respectively. Cyclic voltammetry (CV), electrochemical impedance spectroscopy (EIS), and differential pulse voltammetry (DPV) techniques were performed to examine the electrochemical characteristics of the materials.

S3. Synthesis of zeolitic imidazolate framework (ZIF-8)

In 200 mL of CH_3OH, 5.444 g of $Zn(NO_3)_2 \cdot 6H_2O$ and 3.995 g of 2-MIM were separately dissolved. The solution was mixed and stirred for 20 h at room temperature. The resulting mixture was centrifuged at 9000 rpm for 10 min, washed with 95% ethanol, and dried at 50 °C to obtain ZIF-8 white powder.

S4. Chlorination process of SPCE

The electrodes were cleaned with deionized water and then heated at 50 °C for 3 min. A Ag electrode served as the working electrode, with a Pt wire and Ag/AgCl as the counter and reference electrodes, respectively. A 0.1 M KCl solution was used as the electrolytic solution and chlorination was performed using a CHI6114E electrochemical instrument. After chlorination, the electrodes were rinsed, dried, and stored in a dry box in the dark. The following parameters were used for the chlorination process: Initial E (V) = 1, Sample interval (s) = 0.1, Run time (s) = 20; Quiet time (s) = 0; Scales during run = 1; Sensitivity (A/V) = 1×10^{-5}.

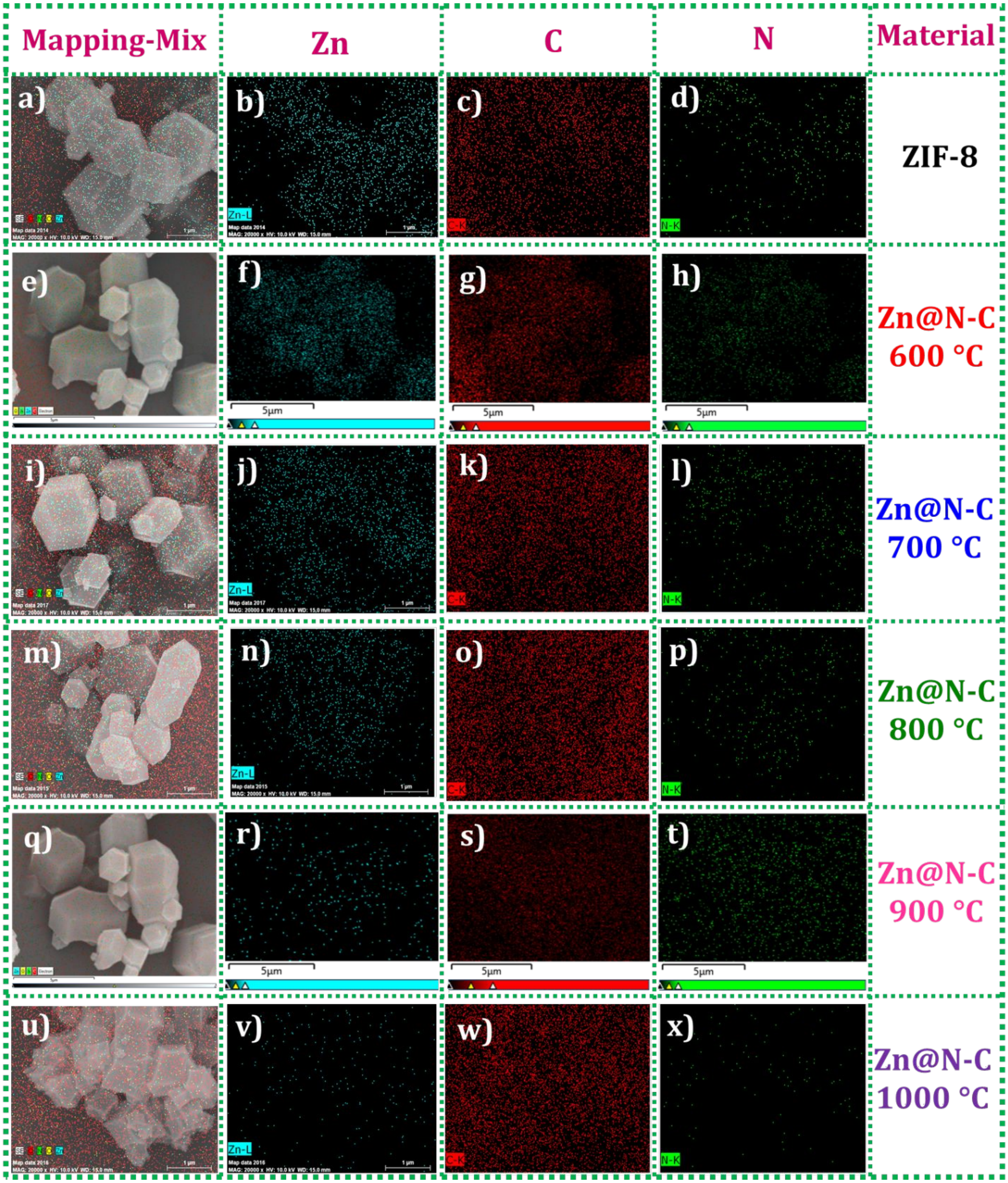

Figure S1. Elemental mapping of (a-d) ZIF-8 and (e-x) Zn@N-C at diverse temperatures (600-1000 °C).

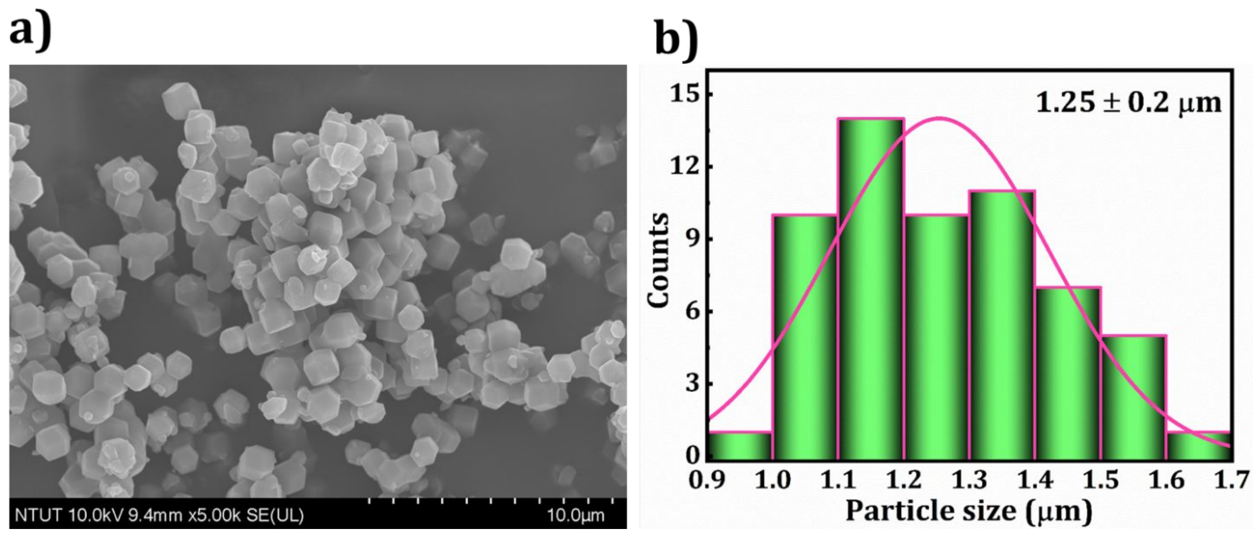

Figure S2. (a) Large-scale FESEM image of Zn@N-C 800 °C with (b) the particle size distribution diagram of Zn@N-C 800 °C.

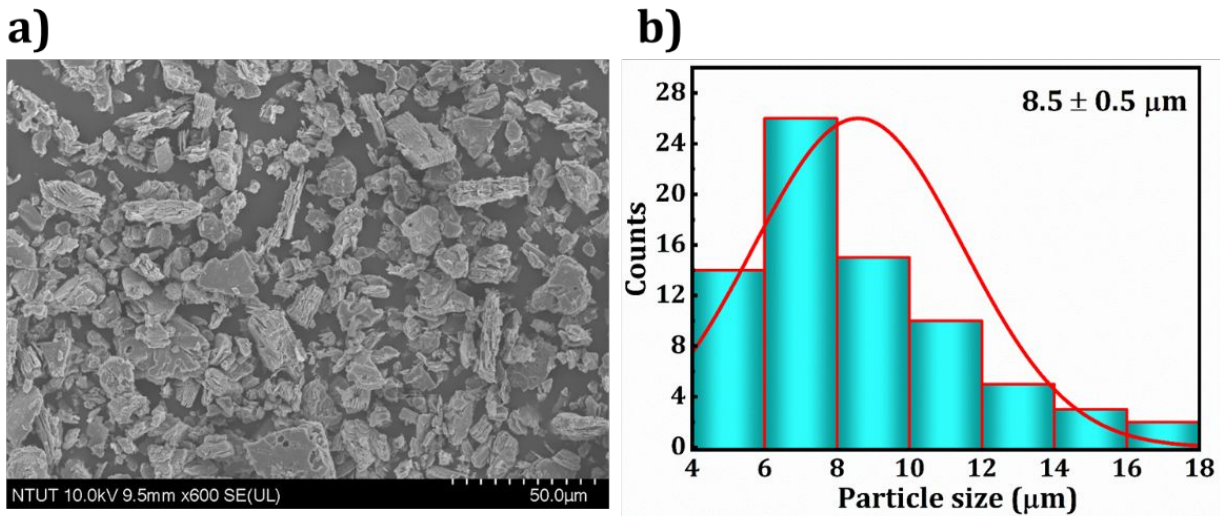

Figure S3. (a) Large-scale FESEM image of MXene with (b) the particle size distribution diagram of MXene.

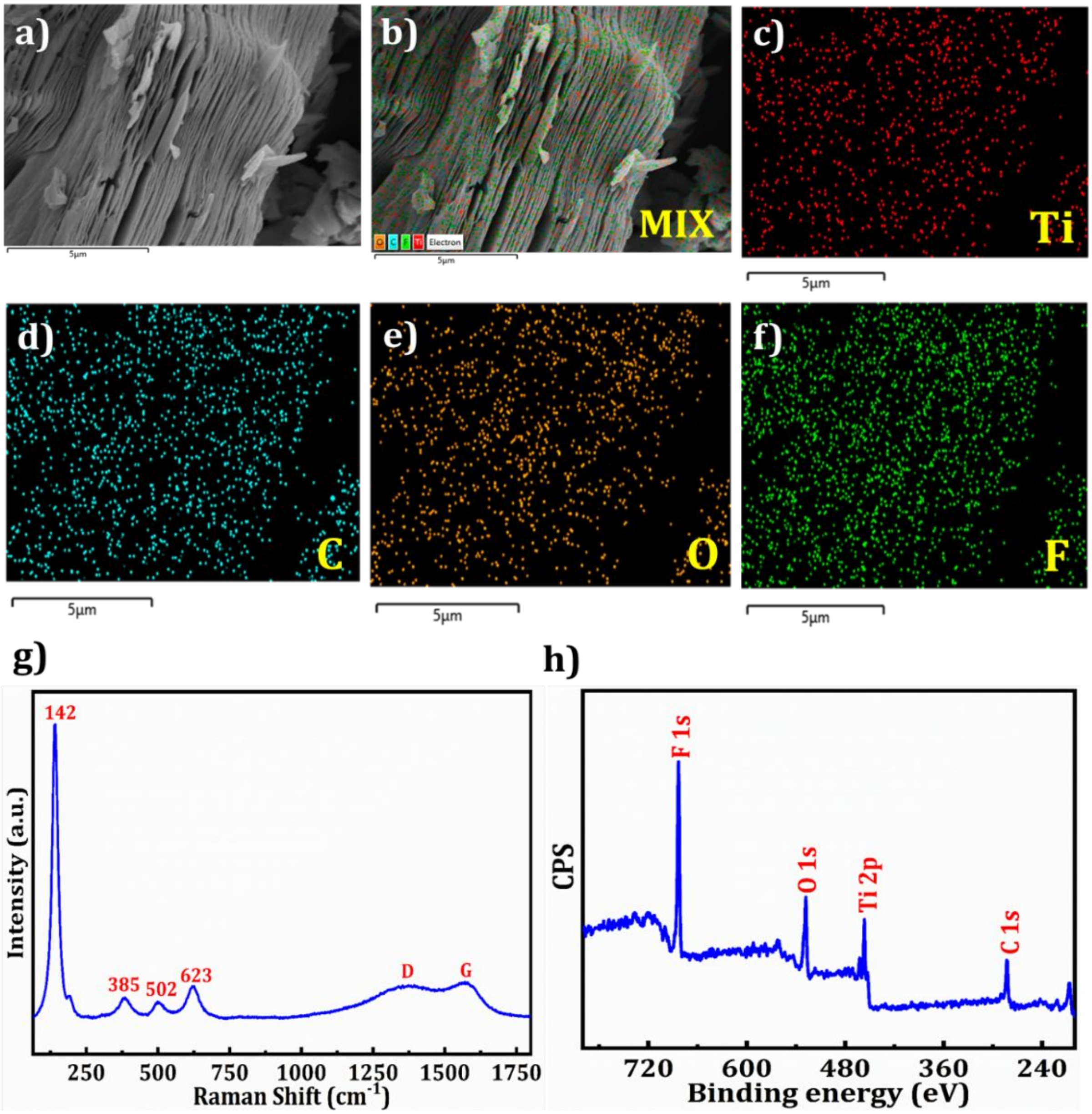

Figure S4. (a) FESEM image, (b-f) elemental mapping of Mix, Ti, C, O, and F, (g) Raman spectrum, and (h) XPS survey spectrum of MXene.

S5. Ultraviolet-visible spectroscopy (UV-Vis) analysis

UV-Vis spectroscopy can be utilized to identify the composition and structure of compounds in solution. Figure S5 displays the UV-Vis spectrum of gold nanostars (AuNSs), which reveals a localized surface plasmon resonance (LSPR) band at a wavelength of 702 nanometers, which is consistent with the previously reported articles [1,2]. This observation confirmed the successful synthesis of AuNSs.

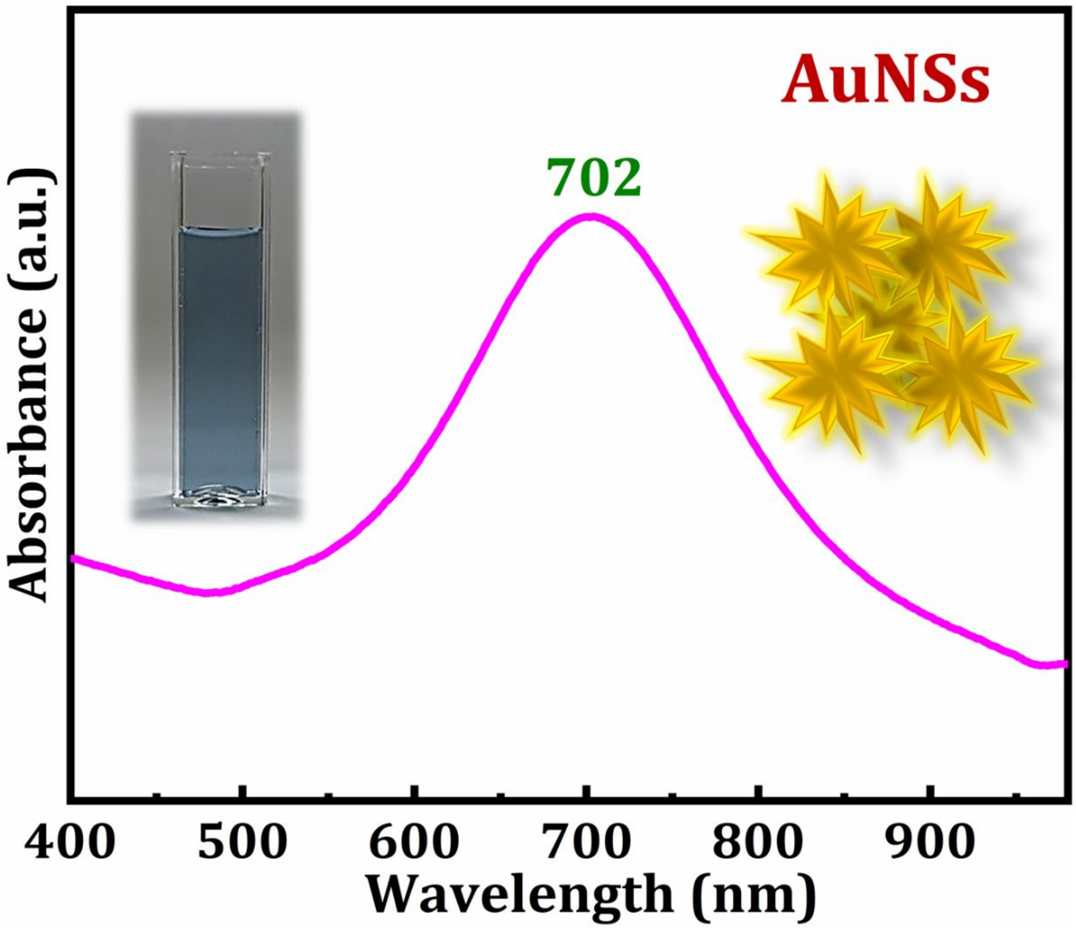

Figure S5. UV-vis spectrum of AuNSs, and the inset picture is the AuNSs solution.

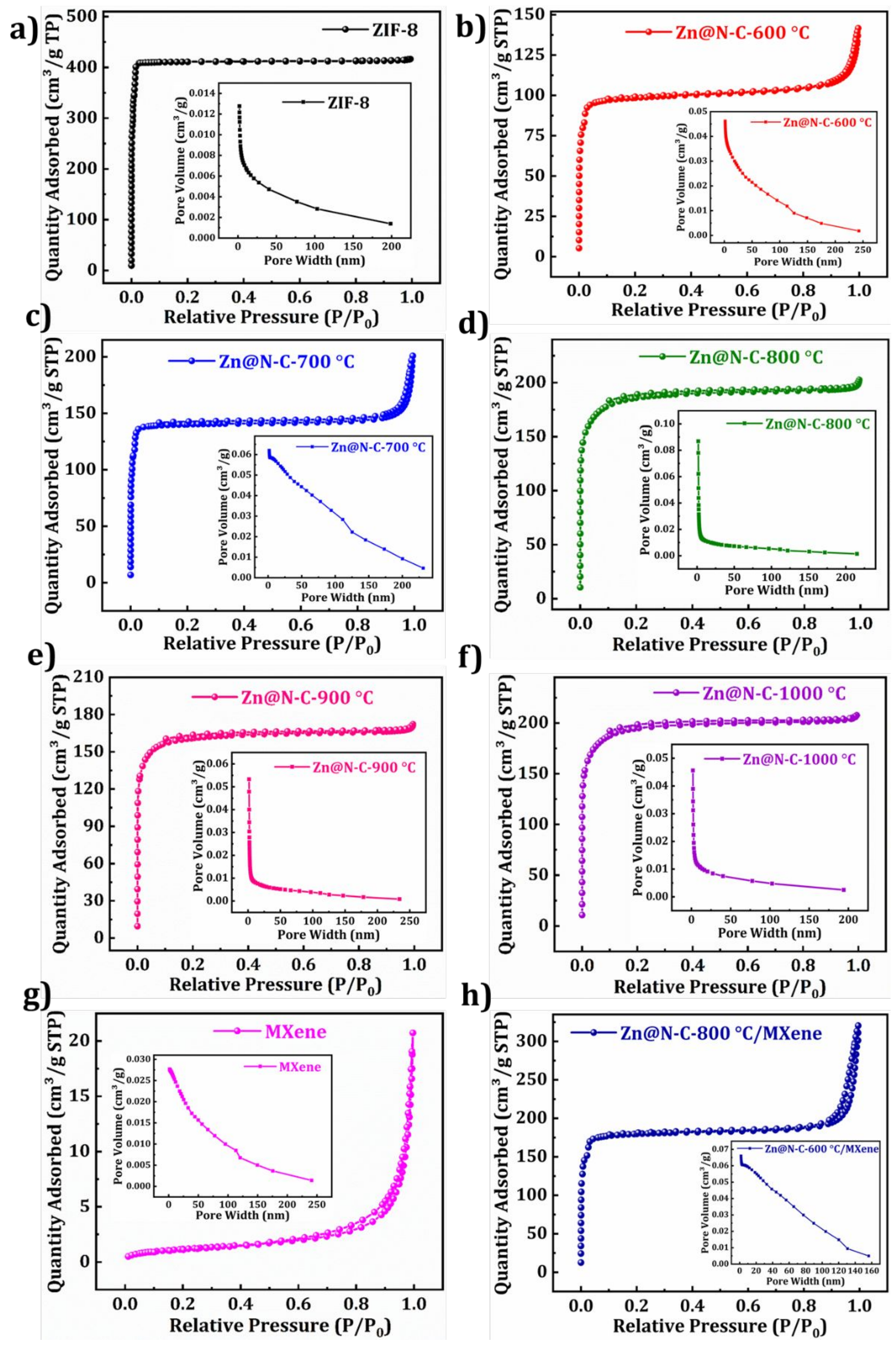

Figure S6. Nitrogen adsorption–desorption isotherms of (a) ZIF-8 and (b-f) Zn@N-C-(600–1000 °C), (g) MXene, and (h) Zn@N-C-800 °C/MXene, and inset: BJH plots.

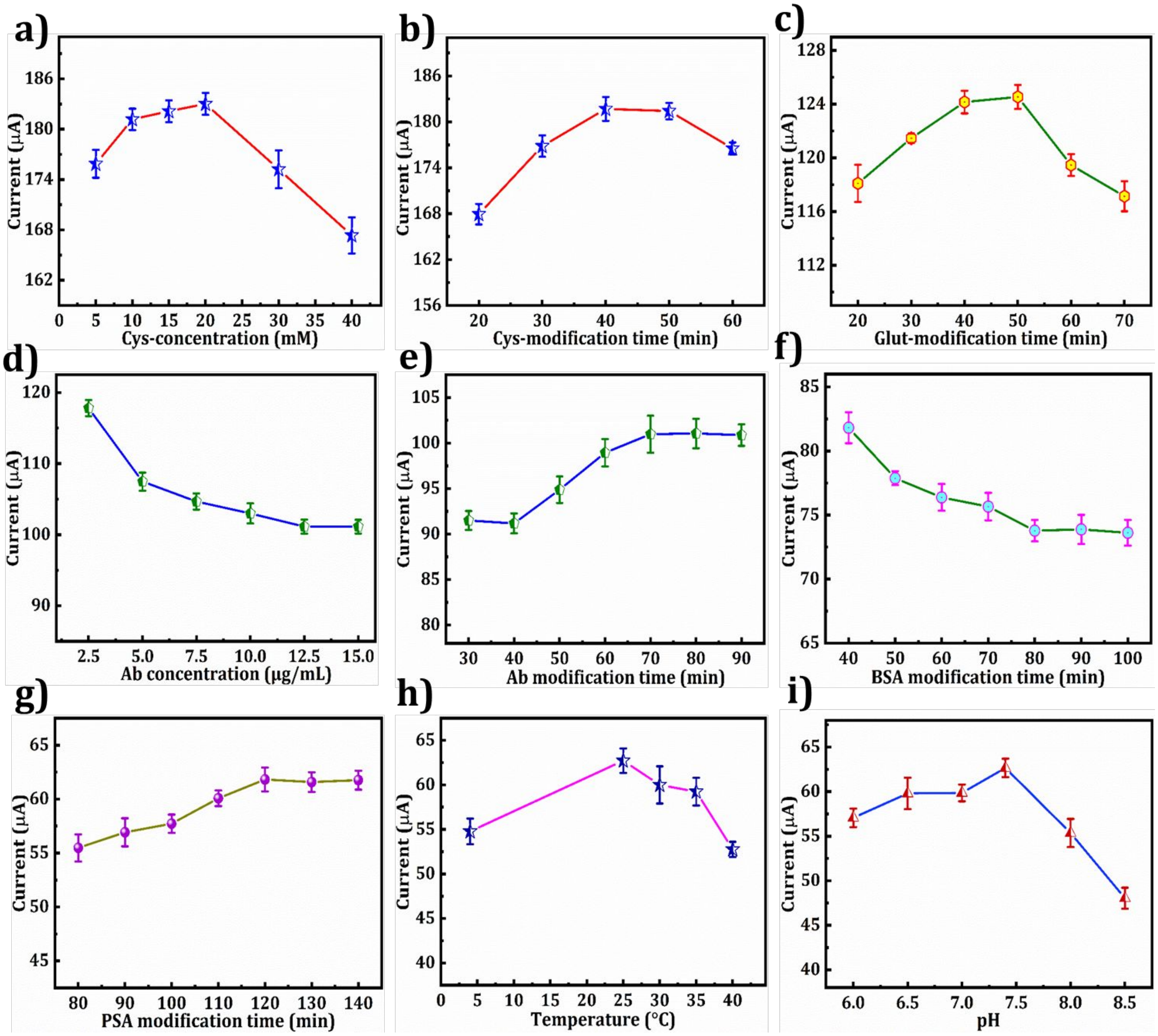

Figure S7. Plots of (a) Cys concentration (from 5 to 40 mM) and (b) Cys modification time (from 0.5 to 5 h) versus current changes. (c) Plot of Glut modification time (from 20 to 70 min) versus current change. Plots of (d) Ab-PSA concentrations (from 2.5 to 15 μg/mL) and (e) Ab-PSA modification time (from 30 to 90 min) versus current changes. (f) Plots of BSA modification time (from 40 to 100 min) versus current change and (g) Antigen PSA modification time (from 80 to 140 min) versus current change. Plots of (h) different temperatures (from 4 to 40 °C) and (i) different pH values (from 6.0 to 8.5) versus current changes.

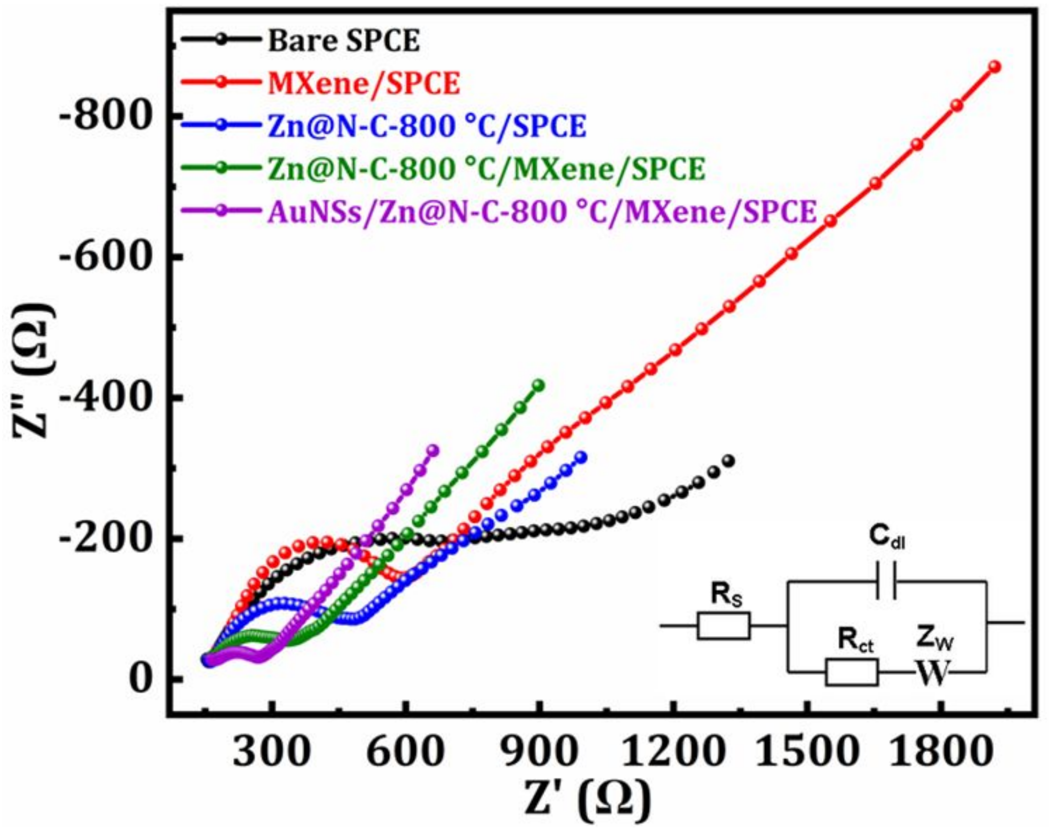

Figure S8. EIS results of bare SPCE, MXene/SPCE, Zn@N-C-800 °C/SPCE, Zn@N-C-800 °C/MXene/SPCE, and AuNSs/Zn@N-C-800 °C/MXene/SPCE.

Table S1. Comparison of different PSA immunosensors.

Material	Technique	linear range	LOD	Reference
AgNPs/rGO/SPCEs	CV	1.0 – 1000 ng/mL	0.01 ng/mL	3
AuNPs/ nano-PEDOT-GA/GCE	DPV	0.0001 – 50 ng/mL	0.03 pg/mL	4
Peptides/Au-rGO/ Au-PMB/GCE	SWV	1 fg/mL – 100 ng/mL	0.11 fg/mL	5
BrPy/AuNP-Hep-Nafion	SWV	0.1 – 50 ng/mL	0.08 ng/mL	6
Bi_2S_3/TiO_2/FTO	Amperometry	1 pg/mL – 100 ng/mL	0.87 pg/mL	7
ce-MoS_2/AgNRs/SPE	CV	0.1 ng/mL – 1000 μg/mL	0.051 ng/mL	8
TB/M-CeO_2/CMC/ILs and Au-CoS/graphene	DPV Amperometry	0.5–50 pg/mL	0.16 pg/mL	9
Thionine/MWCNT/IL	DPV	0.2–40 ng/mL	0.020 ng/mL	10
NiFe PBA/AuNPs	DPV	0.5–1000 pg/mL	0.23 pg/mL	11
g-C_3N_4/CdS:Mn QDs	Amperometry	10–2000 pg/mL	3.80 pg/mL	12
AuNP	DPV	1.0–8.0 ng/ mL	0.55 ng/ mL	13
AuNPs/p(EDOT-co-3MT)/PGE	DPV	0.0001-50 ng/mL .	0.083 pg/mL	14
rGO@SFP	Amperometry	1.7 fM – 1 nM	1.7 fM	15
AuNSs/Zn@N-C-800°C/MXene	DPV	0.1 pg/mL – 1 μg/mL	8.48 fg/mL	**This work**

Abbreviations: AgNPs- gold nanoparticles; rGO- reduced graphene oxide; SPCEs- screen printed carbon electrodes; CV- cyclic voltammetry; nano-PEDOT- poly(3,4-ethylenedioxythiophene); GA-graphene aerogel; GCE – glassy carbon electrode; DPV- differential pulse voltammetry; Au-PMB- Au-poly(methylene blue); SWV- Square wave voltammetry; BrPy-liquid crystal (E)-1-decyl-4-[(4-decyloxyphenyl)diazenyl]pyridinium bromide; Hep- heparin; Bi_2S_3- bismuth sulfide; TiO_2 – titanium dioxide; FTO- fluorine-doped tin oxide; ce-MoS_2- chemical exfoliated molybdenum disulfide; AgNRs-silver nanorods; SPE- screen printed electrode; TB-toluidine blue;M-CeO_2- cerium oxide mesoporous nanoparticles; CMC- carboxymethyl chitosan; ILs- ionic liquids; Au-CoS- gold modified cobalt sulfide; MWCNT- multiwalled carbon nanotube; NiFe PBA- nickel iron prussian blue analogue; g-C_3N_4 – graphitic carbon nitride; CdS:Mn- manganese doped cadmium sulfide quantum dots; p(EDOT-co-3MT)- poly(3,4-ethylenedioxythiophene-co-3-methylthiophene); PGE- pencil graphite electrode; SFP- sunflower pollen particles.

References

(1) Yuan, H.; Khoury, C. G.; Hwang, H.; Wilson, C. M.; Grant, G. A.; Vo-Dinh, T. Gold Nanostars: Surfactant-Free Synthesis, 3D Modelling, and Two-Photon Photoluminescence Imaging. *Nanotechnology* **2012**, *23* (7), 075102. https://doi.org/10.1088/0957-4484/23/7/075102.

(2) Spedalieri, C.; Szekeres, G. P.; Werner, S.; Guttmann, P.; Kneipp, J. Intracellular Optical Probing with Gold Nanostars. *Nanoscale* **2021**, *13* (2), 968–979. https://doi.org/10.1039/D0NR07031A.

(3) Han, L.; Liu, C.-M.; Dong, S.-L.; Du, C.-X.; Zhang, X.-Y.; Li, L.-H.; Wei, Y. Enhanced Conductivity of RGO/Ag NPs Composites for Electrochemical Immunoassay of Prostate-Specific Antigen. *Biosens Bioelectron* **2017**, *87*, 466–472. https://doi.org/10.1016/j.bios.2016.08.004.

(4) Jia, H.; Xu, J.; Lu, L.; Yu, Y.; Zuo, Y.; Tian, Q.; Li, P. Three-Dimensional Au Nanoparticles/Nano-Poly(3,4-Ethylene Dioxythiophene)- Graphene Aerogel Nanocomposite: A High-Performance Electrochemical Immunosensing Platform for Prostate Specific Antigen Detection. *Sens Actuators B Chem* **2018**, *260*, 990–997. https://doi.org/10.1016/j.snb.2018.01.006.

(5) Tang, Z.; Wang, L.; Ma, Z. Triple Sensitivity Amplification for Ultrasensitive Electrochemical Detection of Prostate Specific Antigen. *Biosens Bioelectron* **2017**, *92*, 577–582. https://doi.org/10.1016/j.bios.2016.10.057.

(6) Talamini, L.; Zanato, N.; Zapp, E.; Brondani, D.; Westphal, E.; Gallardo, H.; Vieira, I. C. Heparin-gold Nanoparticles and Liquid Crystal Applied in Label-free Electrochemical Immunosensor for Prostate-specific Antigen. *Electroanalysis* **2018**, *30* (2), 353–360. https://doi.org/10.1002/elan.201700651.

(7) Monteiro, T. O.; dos Santos, C. C.; do Prado, T. M.; Damos, F. S.; Luz, R. de C. S.; Fatibello-Filho, O. Highly Sensitive Photoelectrochemical Immunosensor Based on Anatase/Rutile TiO2 and Bi2S3 for the Zero-Biased Detection of PSA. *Journal of Solid State Electrochemistry* **2020**, *24* (8), 1801–1809. https://doi.org/10.1007/s10008-020-04637-8.

(8) Gui, J.-C.; Han, L.; Du, C.-X.; Yu, X.-N.; Hu, K.; Li, L.-H. An Efficient Label-Free Immunosensor Based on Ce-MoS2/AgNR Composites and Screen-Printed Electrodes for PSA Detection. *Journal of Solid State Electrochemistry* **2021**, *25* (3), 973–982. https://doi.org/10.1007/s10008-020-04872-z.

(9) Wei, Y.; Li, X.; Sun, X.; Ma, H.; Zhang, Y.; Wei, Q. Dual-Responsive Electrochemical Immunosensor for Prostate Specific Antigen Detection Based on Au-CoS/Graphene and CeO2/Ionic Liquids Doped with Carboxymethyl Chitosan Complex. *Biosens Bioelectron* **2017**, *94*, 141–147. https://doi.org/10.1016/j.bios.2017.03.001.

(10) Salimi, A.; Kavosi, B.; Fathi, F.; Hallaj, R. Highly Sensitive Immunosensing of Prostate-Specific Antigen Based on Ionic Liquid–Carbon Nanotubes Modified Electrode: Application as Cancer Biomarker for Prostatebiopsies. *Biosens Bioelectron* **2013**, *42*, 439–446. https://doi.org/10.1016/j.bios.2012.10.053.

(11) Hua, X.; Zhao, T.; Gui, X.; Jin, B. A NiFe PBA/AuNPs Nanocomposite Sensitive Immunosensor for Electrochemical Detection of PSA. *Analytical Methods* **2024**, *16* (13), 1923–1933. https://doi.org/10.1039/D3AY02184J.

(12) Zhang, K.; Lv, S.; Lin, Z.; Tang, D. CdS:Mn Quantum Dot-Functionalized g-C3N4 Nanohybrids as Signal-Generation Tags for Photoelectrochemical Immunoassay of Prostate Specific Antigen Coupling DNAzyme Concatamer with Enzymatic Biocatalytic Precipitation. *Biosens Bioelectron* **2017**, *95*, 34–40. https://doi.org/10.1016/j.bios.2017.04.005.

(13) de Oliveira Cândido, T. C.; Pereira, A. C.; da Silva, D. N.; Ferreira, L. F.; Tarley, C. R. T. Development of a Screen-Printed Electrochemical Immunosensor Modified with Gold Nanoparticles for Prostate-Specific Antigen (PSA) Detection. *Journal of Solid State Electrochemistry* **2024**. https://doi.org/10.1007/s10008-024-05939-x.

(14) Uruc, S.; Dokur, E.; Gorduk, O.; Sahin, Y. Disposable and Ultrasensitive Label-Free Gold Nanoparticle Patterned Poly(3,4-Ethylenedioxythiophene- *Co* -3-Methylthiophene) Electrode for Electrochemical Immunosensing of Prostate-Specific Antigen. *New Journal of Chemistry* **2024**, *48* (23), 10415–10426. https://doi.org/10.1039/D4NJ02034K.

(15) Wang, L.; Jackman, J. A.; Ng, W. B.; Cho, N. Flexible, Graphene-Coated Biocomposite for Highly Sensitive, Real-Time Molecular Detection. *Adv Funct Mater* **2016**, *26* (47), 8623–8630. https://doi.org/10.1002/adfm.201603550.
